# Modulation of the functional association between the HIV-1 intasome and the nucleosome by histone amino-terminal tails

**DOI:** 10.1186/s12977-017-0378-x

**Published:** 2017-11-28

**Authors:** Mohamed S. Benleulmi, Julien Matysiak, Xavier Robert, Csaba Miskey, Eric Mauro, Delphine Lapaillerie, Paul Lesbats, Stéphane Chaignepain, Daniel R. Henriquez, Christina Calmels, Oyindamola Oladosu, Eloïse Thierry, Oscar Leon, Marc Lavigne, Marie-Line Andreola, Olivier Delelis, Zoltán Ivics, Marc Ruff, Patrice Gouet, Vincent Parissi

**Affiliations:** 1 Fundamental Microbiology and Pathogenicity Laboratory, UMR 5234 CNRS-University of Bordeaux, SFR TransBioMed, 146 rue Léo Saignat, Bordeaux Cedex, France; 2 MMSB-Institute of the Biology and Chemistry of Proteins, UMR 5086 CNRS-Lyon 1 University, Lyon, France; 3 Division of Medical Biotechnology, Paul Ehrlich Institute, Langen, Germany; 4 UMR CNRS 5248 CBMN (Chimie Biologie des Membranes et Nanoobjets), Université de Bordeaux, 33076 Bordeaux, France; 5 Virology Program, ICBM, Faculty of Medicine, University of Chile, Santiago of Chile, Chile; 6 Département de Biologie Structurale Intégrative, UDS, U596 INSERM, UMR7104 CNRS, IGBMC (Institut de Génétique et de Biologie Moléculaire et Cellulaire), Illkirch, France; 7 LBPA, UMR8113, CNRS, ENS-Cachan, 94235 Cachan, France; 8 Dpt de Virologie, UMR 3569, CNRS, Institut Pasteur, Paris, France; 9 Institut Cochin-Inserm U1016-CNRS UMR8104-Université Paris Descartes, Paris, France; 10 International Associated Laboratory (LIA) of Microbiology and Immunology, CNRS, University de Bordeaux/Heinrich Pette Institute-Leibniz Institute for Experimental Virology, Bordeaux, France; 11 Viral DNA Integration and Chromatin Dynamics Network (DyNAVir), Bordeaux, France

**Keywords:** Retroviral integration, HIV-1, Integrase, Chromatine, Nucleosome, Histone tails

## Abstract

**Background:**

Stable insertion of the retroviral DNA genome into host chromatin requires the functional association between the intasome (integrase·viral DNA complex) and the nucleosome. The data from the literature suggest that direct protein–protein contacts between integrase and histones may be involved in anchoring the intasome to the nucleosome. Since histone tails are candidates for interactions with the incoming intasomes we have investigated whether they could participate in modulating the nucleosomal integration process.

**Results:**

We show here that histone tails are required for an optimal association between HIV-1 integrase (IN) and the nucleosome for efficient integration. We also demonstrate direct interactions between IN and the amino-terminal tail of human histone H4 in vitro. Structure/function studies enabled us to identify amino acids in the carboxy-terminal domain of IN that are important for this interaction. Analysis of the nucleosome-binding properties of catalytically active mutated INs confirmed that their ability to engage the nucleosome for integration in vitro was affected. Pseudovirus particles bearing mutations that affect the IN/H4 association also showed impaired replication capacity due to altered integration and re-targeting of their insertion sites toward dynamic regions of the chromatin with lower nucleosome occupancy.

**Conclusions:**

Collectively, our data support a functional association between HIV-1 IN and histone tails that promotes anchoring of the intasome to nucleosomes and optimal integration into chromatin.

**Electronic supplementary material:**

The online version of this article (10.1186/s12977-017-0378-x) contains supplementary material, which is available to authorized users.

## Background

Retroviral integrases (INs) are key enzymes that catalyze the insertion of viral DNA into infected cells genome (for a recent review see [1]). Integration occurs in strongly preferred regions of the genome that depend on the virus. Although the IN is a major viral determinant in the integration site selection [2], cellular targeting factors such as BET or LEDGF/p75 proteins, which bind specific histone marks, also contribute to this process by interacting with the IN·viral DNA complex (i.e., the intasome) in these specific chromatin regions (reviewed in [3]). Additional parameters, such as the nuclear import pathway, the nuclear architecture and the interaction of cellular factors like CPSF6 with other viral components, also affect retroviral integration selectivity [4]. Thus, integration site selection is a multi-step process that first involves a global targeting of the intasome toward a suitable chromatin region via the association between IN and cellular factors, followed by local insertion step requiring IN-nucleosome interaction.

This final association between IN and its nucleosomal target substrate is a process governed by the intasome and nucleosomal DNA constraints and regulated by nucleosome density and remodeling activities [5–8]. Indeed, the data from the literature also indicate that while HIV-1 integration occurs at the surface of the nucleosomes, their compaction into dense chromatin limits efficient integration [6, 8]. We have previously shown that chromatin remodeling processes overcome this integration inhibition and favor HIV-1 integration [8]. Furthermore, we have recently reported that local nucleosome dissociation by the FACT histone chaperon generates chromatin structures favoring HIV-1 integration both in vitro and in cells [9]. Taken together these data suggest that additional contacts between the HIV-1 intasome and the nucleosome, which may be prevented during compaction and made accessible during chromatin remodeling, could be required for efficient integration. This hypothesis is supported by the cryoEM structure of the PFV intasome in complex with a mononucleosome showing direct interactions between IN protomers and histones [10]. Moreover, integration assays performed on DNA mini-circles (MCs) mimicking the nucleosomal DNA structure in the absence of histones also suggested that both this structure and additional IN/histone interactions can act in synergy during nucleosomal integration [11]. Consequently, due to the lack of information regarding the mechanisms of nucleosome capture by the HIV-1 intasome, we investigated the potential role of IN/histone interactions in regulating HIV-1 integration.

Using various biochemical and cellular approaches, we show that histone tails are required for efficient HIV-1 IN binding to nucleosomes and optimal integration. We also report that IN binds preferentially to the amino-terminal peptide tail of histone H4 (H4) in vitro and this binding is required for efficient functional interaction between the intasome and the nucleosome. Mutations affecting the IN/histone tail interaction also affect the integration step in cells. Consequently, our data lead us to conclude that the direct interaction between HIV-1 IN and histone tails may facilitate the tethering of the retroviral intasome to the nucleosomes for efficient integration into the host genome.

## Results

### Amino-terminal histone tails modulate the interaction between HIV-1 IN and the nucleosome in vitro

**Fig. 1:**
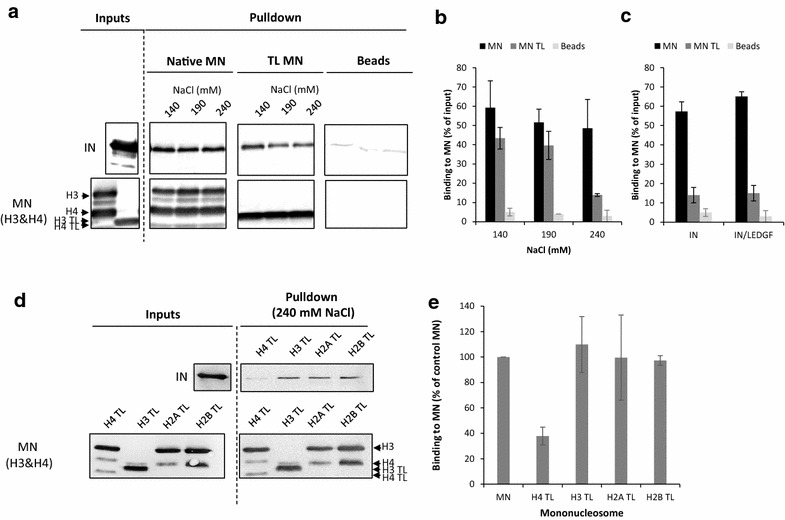
Functional interaction between HIV-1 IN and native or tailless mononucleosomes. Pull-down experiments were performed using WT IN (10 pmol) and either recombinant 601 native mononucleosomes (Native MN) or tailless MNs (TL MN) (125 ng in DNA) at 140, 190 and 240 mM NaCl concentration (lanes 140, 190 and 240). Precipitated IN was detected by western blotting using a polyclonal anti-IN antibody (IN), MNs were detected using a mixture of anti-histone H3 or H4 antibodies (MN H3&H4) (see representative pull down assay in **a**). The bound IN was quantified and reported as the percentage of input precipitated under each condition. Interactions between IN and native or tailless MN at 140–240 ranged NaCl concentration are reported in (**b**). Interactions between the IN/LEDGF complex (10 pmol of IN) and the native or tailless MN at 240 mM NaCl are reported in (**c**). Interactions between IN and the MN deleted either for their H4, H3, H2A or H2B tail (lanes H4 TL, H3 TL, H2A TL and H2B TL) are shown in **(d)** and quantification in (**e**). All values are shown as the mean ± standard deviation (error bars) of three independent sets of experiments. Unspecific interactions between IN or IN/LEDGF complex and beads without MN are also reported (**a**–**c**)

To determine whether the presence of histone tails was required for the association between HIV-1 IN and the nucleosome, we performed in vitro pull-down experiments using recombinant purified IN and either native human mononucleosomes (MNs) or tailless MNs (TL MNs) assembled on the previously described 147-bp W601 Widom sequence [12] biotinylated on its 5′ end (see the MN assembly analysis in Additional file 1: Figure S1). As shown in Fig. 1, IN exhibited different affinities for native MNs and TL MNs. Indeed, increasing salt concentrations decreased the association between IN and TL MNs more efficiently than the association between IN and native MNs (Fig. 1a, b). Similar results were obtained with the IN·LEDGF/p75 complex, indicating that this functional complex also required the presence of native tails for optimal association with the nucleosome (Fig. 1c). To better determine the contribution of each histone tail in the IN/MN binding, we next performed pull-down experiments with MNs assembled using octamers lacking the tails of either H4, H3, H2A or H2B. As shown in Fig. 1d, e, the efficiency of IN binding to the H4TL MNs was approximately 50–60% less efficient than for the native and other MN variants. Interestingly, the deletion of all the histone tails had a larger impact on IN/MN binding than deletion of the H4 tail only. This may indicate that several histone tails could participate together in the binding process, the histone H4 tail appearing the most important protein determinant of this binding. To further determine the impact of histone tails on active IN/viral DNA intasomes, we next performed functional integration assays using the different MN variants.

### Amino-terminal histone tails modulate the integration into nucleosomes catalyzed by HIV-1 IN in vitro

**Fig. 2:**
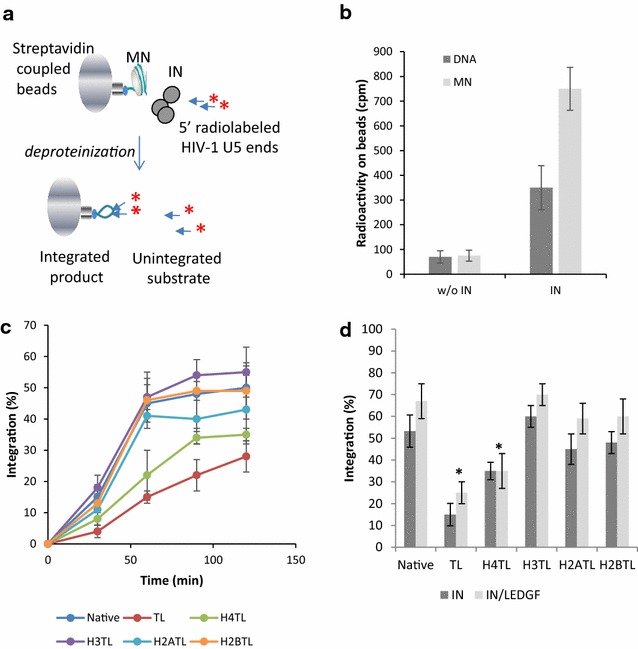
In vitro Integration onto mononucleosomes. Either the 5′ biotinylated naked 601 DNA fragment or the native MNs (50 ng in DNA) were coupled to streptavidin beads and incubated with HIV-1 WT IN (400 nM) under integration conditions reported in the "Methods" section (**a**). After 0–2 h incubations the samples were deprotenized and washed after beads magnetization, then radioactivity was measured on both the pellet and supernatant. Quantification of the radioactivity remaining on beads after reaction performed with naked 601 DNA or MN and with or without IN is reported (**b**). The percentage of integrated product over time for each MN construct was reported in (**c**). Comparison of data obtained with IN alone and IN/LEDGF complex is reported in (**d**). All values are shown as the mean ± standard deviation (error bars) of three to four independent sets of experiments. The p values were calculated by Student’s t-test and are shown as *p < 0.05 to represent the probability of obtaining significant differences compared with the data obtained with the native MNs control

The impact of histone tails on integration activity was then evaluated in in vitro integration assays. For this purpose, the quantitative assay schematized in Fig. 2a was set up using MNs immobilized on streptavidin beads, recombinant IN and a viral DNA donor carrying the 40/42 final base pairs of the HIV-1 U5 sequence (see the "Methods" section for the description of the donor DNA). Optimized reaction conditions set up in the presence of PEG and DMSO (see materials and methods section) were first used to allow analysis of IN activity in the absence of LEDGF. The quantification of the radioactivity that remained on the beads after the reaction, washing and deproteinization, allowed us to quantify the integration efficiency. Control experiments first showed that viral DNA integrated more efficiently into MNs than into naked DNA (Fig. 2b). This result confirmed very early data reporting that MNs are the preferred substrate for HIV-1 integration [13, 14] and validated our system. Integration kinetics experiments showed that viral DNA integrated less efficiently into TL MNs than into native MNs (Fig. 2c). Speed and efficiency of integration were also decreased when H4TL MNs were used, but to a lesser extent. Notably, integration efficiency was found to be lower when using TL MNs than when using H4TL MNs, suggesting that several histone tails could act in concert for optimal integration as suggested by the binding data. Deletion of the H3 tail slightly increased the integration efficiency, while deletion of the tails of other histone variants had no significant effect on the global integration efficiency. The presence of LEDGF/p75 did not alter the effect of histone tail deletion on integration under these conditions (Fig. 2d) and even when non-optimized reactions allowing a maximal LEDGF/p75 stimulatory effect were used (i.e. without PEG and DMSO, Additional file 1: Figure S2).

Taken together, these data indicate that native amino-terminal histone tails are required for optimal IN binding to MNs and efficient integration in vitro. Binding experiments between IN and histone tails were next performed to further investigate whether this integration modulation could be due to such direct interactions.

### Interaction between HIV-1 IN and histone amino-terminal peptide tails

**Fig. 3:**
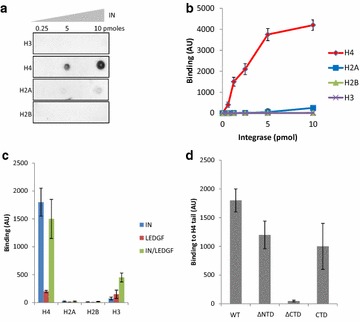
FAR dot-blot analysis of the interactions between HIV-1 IN and peptides derived from histones amino-terminal tails. The associations between IN and H3, H4, H2A and H2B biotinylated peptides from the histones tails (sequences in Figure S3) were evaluated using a far dot blot approach as described in the "Methods" section using 1 µl of 0.25 − 10 pmol of recombinant IN (lanes 0.25, 5 and 10) spotted onto a nitrocellulose membrane and 1 µM of peptide H3, H4, H2A or H2B (a typical result is shown in **a**). The far dot blots were run three to ten times and the intensity of each spot was quantified using ImageJ software. The results are reported as the mean of the experiments ± standard deviation (**b**). Same experiments were conducted using IN, LEDGF/or the IN/LEDGF complex and results obtained with 2.5 pmol of the different proteins are reported in (**c**). The far dot blot assays were performed to identify the HIV-1 IN domain responsible for the recognition of the H4 histone tail. 2.5 pmol of truncated proteins lacking the NTD (∆NTD) or the CTD (∆CTD), or the isolated CTD (CTD), immobilized together with full-length WT IN were incubated with the H4 tail. Binding was quantified, and the results are represented as the mean of three to six independent experiments ± standard deviation in (**d**)

Possible direct interactions between HIV-1 IN and histone tails were analyzed using a far dot blot approach with recombinant IN and peptides derived from the H3, H4, H2A and H2B amino-terminal tail (see peptide sequences in Additional file 1: Figure S3). As reported in Fig. 3a and quantification in b, interaction was significantly detected only in the presence of the histone H4 tail. Similar results were obtained with the purified IN·LEDGF/p75 complex, indicating that the LEDGF/p75 cofactor did not affect IN binding to the peptide (Fig. 3c). Additional analyzes showed that the IN/H4 tail interaction could be negatively or positively modulated by amino acid modifications as methylation of K20 or K20 or K16 acetylation (Additional file 1: Figure S4).

The far dot blot approach was then adapted to compare different IN truncation mutants in order to identify the IN domains involved in the interaction to H4 tail. Under these conditions, the engineered IN 50–288 amino acid construct lacking the amino-terminal domain (∆NTD) and the isolated 220–288 amino acid CTD domain construct (CTD) show similar binding properties when compared to the wild-type (WT) enzyme (Fig. 3d). By contrast, the association with the histone H4 tail was almost completely abolished for the 1-212 amino acid construct lacking the carboxy-terminal domain (∆CTD). These results show that the CTD domain is responsible for the interaction between IN and histone tail. In order to study the role of this interaction in the integration process we further searched for specific amino-acids mutations that could affect the IN binding to the tail.

### Identification of IN mutations affecting the binding to histone H4 tail

**Fig. 4:**
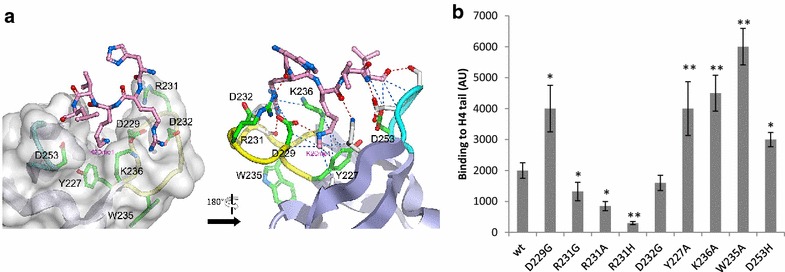
Identification of amino-acids positions modulating the IN/H4 interaction. The interaction between the HIV-1 CTD and a pentapeptide derived from the H4K20me1 modified histone tail was predicted from docking simulations. The representation of the H4K20me1 pentapeptide (pink ball-and-stick model) docked into the HIV-1 IN CTD (gray surface) is shown in (**a**). The 228-235 and 253-257 loops are shown in yellow and cyan, respectively. Residues Y227, R231 and W235, represented in stick form, are highlighted in green. The model shows the K20me1 side chain pointing down into the V-shaped groove defined by loops 228–235 and 253–257. View of the docking model rotated 180° relative to panel A, using the same color scheme. Predicted hydrogen bonds and hydrophobic contacts are depicted by red and blue dashed lines, respectively. Residues interacting with the H4K20me1 pentapeptide are depicted by white sticks. Residues highlighted in green are those being mutated in this study. At the exception of W235, they all interact with the pentapeptide as well. Point mutations were introduced at residues potentially involved in H4 tail interaction recognition and their binding to the histone H4 peptide tail was analyzed using far dot blot experiments (**b**, see text for details). The binding measured with 5 pmol of enzyme is reported as the mean of three to ten independent experiments ± standard deviation. The p values were calculated by Student’s t-test and are represented as *p < 0.05 and **p < 0.005 to denote the probability of obtaining significant differences compared with the data obtained with the WT enzyme

We first adopted an in silico blind docking simulation approach starting from a fragment spanning residues 210–270 from the 2.8 Å resolution HIV-1 IN CCD-CTD structure [15] and a pentapeptide mimicking the 18–22 residues from the H4K20me1-modified histone (H_18_RK_me_VL), which corresponds to the best IN binder in the previous analyzes (see Additional file 1: Figure S4). In the first set of experiments, the AutoDock and AutoDockVina programs were used in parallel to determine a potential binding region based on a blind docking analysis of the entire surface of the receptor, namely, the IN CTD fragment, which was treated as rigid. Following a cluster analysis of all docked conformations computed by AutoDock, a potential binding site emerged in the HIV-1 IN CTD encompassing a V-shaped groove area delineated by loops 228–235 and 253–257 (one connecting strands β1 and β2 and the other connecting β3 and β4, respectively) (Fig. 4a). The resulting docking solution is compatible with the 3.9 Å resolution cryoEM structure of the HIV-1 strand transfer complex (STC) intasome [16], in which the V-shaped CTD grooves are accessible in all the assembled IN protomers.

To determine the IN residues that may be involved in the CTD-H4 tail interaction, we focused on this latter region, where several amino acid side-chains surrounding the V-shaped groove of the receptor were treated as flexible (namely, Y227, D229, S230, R231, D232, L234, W235, K236, D253, N254, S255, D256, K258 and K264). RMSD cluster analysis of 1000 independent docking solutions using the AutoDock program allowed 56 distinct conformational groups to be defined. Considering the binding energies one solution stood out in particular, where the peptide was engaged in a total of 7 intermolecular hydrogen bonds (with the side-chains of D229, R231, S255, D256, and K258 and the backbone of L234 in the HIV-1 CTD) and 15 hydrophobic contacts (with the side-chains of Y227, D229, D232, K236, D256, K258 and V260 and the backbones of D229, S255 and D256). In this model, the peptide adopted an elongated shape at the surface of the IN CTD, with the H4K20me1 side-chain pointing down into the V-shaped groove, and formed 9 of the 16 predicted hydrophobic contacts (involving Y227, D229, K236, K258 and V260 HIV-1 CTD amino-acids residues) as well as one hydrogen bond (with D229) (Fig. 4a). Slight side-chain movements were observed to accommodate the pentapeptide, with the exception of R231 IN residue, whose side-chain flipped to form a hydrogen bond with H18 from histone 4 tail. This model was used to design a site-directed mutagenesis approach. The CTD domain has been shown to be involved in multiple functions during the viral life cycle, including interactions with reverse transcriptase and target DNA [17–19]. This made it difficult to generate CTD mutants that only affected histone binding. We focused on amino acids Y227, D229, R231, W235, K236 and D253, which were expected (1) to be located in the V-shaped groove of the IN CTD and (2) to be involved directly or indirectly in modulating the interaction. Alanine, glycine or histidine substitutions were introduced at the chosen positions to test peptide binding. The D232G substitution was also included because it represents a natural polymorphism in HIV-1 IN.

All mutants were purified, and their overall functional structures were examined in in vitro concerted integration assay. As shown in Additional file 1: Figure S5, the Y227A and W235A mutations severely affected integration (90–70% loss of activity). The K236A and D229G mutations also influenced IN catalysis, but to a lesser extent (20–40% loss). By contrast, the D232G, R231G/A/H and D253H proteins were fully active. A far dot blot assay was the used to determine the ability of the mutants to bind to and recognize the histone H4 tail. The R231G/A/H mutants showed a decrease in their overall binding to the H4 amino-terminal tail (30, 44 and 77%, respectively; Fig. 4b). Additionally, the binding properties of the D232G mutant were virtually unaffected, whereas D229G showed a global increase in H4 tail affinity. Conversely, the Y227A, W235A, K236A and D253H mutants displayed a significant increase in affinity for the histone H4 tail.

In summary, most of the designed mutations, except the natural D232G variant, significantly affected the IN binding to the H4 tail suggesting that the corresponding amino-acids position modulate the IN/H4 interaction directly or indirectly. The identified mutants were then used to further investigate the role of the IN/H4 interaction in the association with nucleosomes.

### Effect of mutations affecting IN binding to H4 on the functional interaction with nucleosomes in vitro

To avoid any biases in the analysis of the MN-binding properties of the mutated INs due to the alteration of IN-DNA interaction, we first evaluated their DNA-binding properties by pull-down experiments using the naked W601 fragment. The Y227A, W235A and K236A mutants each showed decreased affinity for DNA (Additional file 1: Figure S6), which correlates well with their relative levels of in vitro integration activity. Consequently, we excluded these enzymes from the MN interaction studies, and the mutants that showed unaffected DNA-binding capability were further analyzed for their capacity to associate with MNs.

**Fig. 5:**
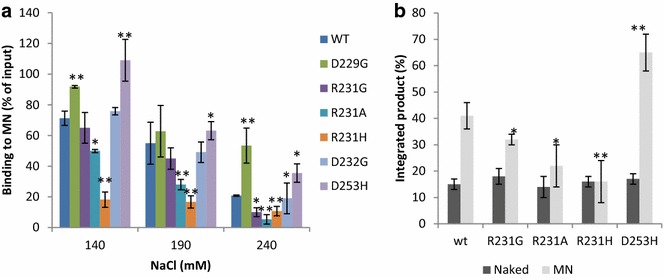
Effect of IN/H4 mutations on the functional association between HIV-1 IN and mononucleosomes. Pull-down experiments were performed using recombinant 601 mononucleosome (125 ng in DNA) and WT IN or mutant proteins (10 pmol) under 140–240 mM NaCl (see typical experiments in Figure S5). Bound IN was detected by western blotting using a polyclonal anti-IN antibody and quantified as reported in (**a**) as the percentage of input precipitated under each condition. Integration assays were performed on MN (50 ng in DNA) immobilized on streptavidin beads with 400 nM of WT or mutated IN and 10 nM of 42 bp of a 5′-radiolabeled viral U5 end. The percentage of integrated product was measured as indicated in materials and methods section and is shown in (**b**). All values are shown as the mean ± standard deviation (error bars) of three to six independent sets of experiments. The p values were calculated by Student’s t-test and are shown as *p < 0.05 and **p < 0.005 to represent the probability of obtaining significant differences compared with the data obtained with the WT enzyme

As shown in Fig. 5a (see detailed analysis in Additional file 1: S6), the R231A/H mutants showed a significant decrease in MN binding affinity, which parallels their reduced affinity for the histone tail. The R231G mutant also had a decreased affinity for MN, but to a lesser extent, as a significant decrease in IN/MN binding was detected only at NaCl concentrations above 190 mM. By contrast, the D229G and D253H mutants, which showed an increased affinity for the H4 histone tail, also showed increased binding to MNs. The MN-binding capabilities of the natural D232G variant were not significantly affected. We next tested the effect of the mutations on the catalysis of integration into nucleosomes.

In vitro integration assays were performed using the recombinant W601 MNs used in the pull-down experiments (Fig. 5b). Control experiments performed with the unassembled W601 DNA fragment confirmed that the ability of the mutants to catalyze integration into naked DNA was not affected. In contrast, the R231G/A/H IN mutants exhibited a 25–60% decrease in efficiency of integration into MNs, and the D253H mutant was 20–40% more active than the WT enzyme. This result finely correlates with the capability of the different INs to bind the H4 tail/MNs and fully supports our hypothesis that the binding to the tail is required for optimal integration into MNs in vitro. Therefore, we next investigated the impact of this IN/H4 interaction in a viral context.

### Effect of IN/H4 mutations on viral infectivity and integration efficiency

**Fig. 6:**
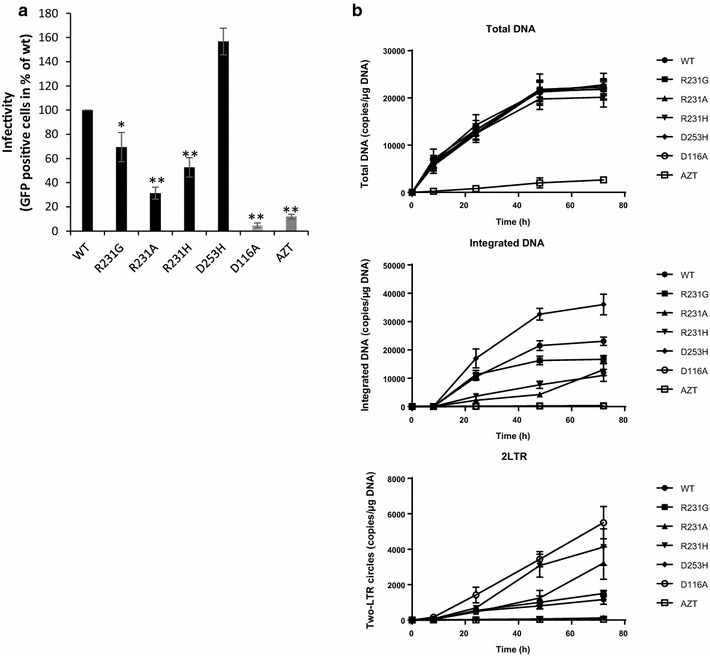
Effects of mutations affecting the IN/H4 interaction during early steps of viral replication. HEK-293T cells were transduced with VSV-G pseudotyped lentiviruses encoding either WT IN or the R231A/H/G or D253H IN mutants or the catalytically inactive class I D116A mutant with or without AZT 1 µM. Viral replication was quantified based on eGFP fluorescence measured by FACS 48 h post-transduction. The data shown in (**a**) are expressed as the percentage of eGFP-positive cells at a MOI of 1. The replication steps affected by the mutations were determined by measuring the amounts of the different viral DNA species produced using qPCR. Levels of total viral DNA, integrated DNA and 2-LTR circles shown respectively in (**b**) were monitored between 0 and 72 h post-transduction to check for potential defects at the steps of reverse transcription, integration and nuclear import of the preintegration complex, respectively. The data are represented as the mean of at least three independent experiments ± standard deviation. The p-values were calculated by Student’s t-test and are shown as *p < 0.05 and **p < 0.005 to represent the probability of obtaining significant differences compared with the WT data

Retroviral vectors carrying the selected R231G/A/H and D253H IN mutations, which modified the IN/H4 interaction without affecting the intrinsic IN catalytic properties, were produced, and their early replication steps were examined. The infectivity of the mutants was compared to that of WT vectors using a single-round infection assay performed in 293T cells. As shown in Fig. 6a, the infectivity of the R231G/A/H viruses was reduced by 20, 40 and 60% when compared with the WT virus, respectively. By contrast, the D253H mutation showed a 40–60% increase in viral infectivity.

The replication stages affected by the mutations were further characterized by comparing the viral DNA population size of the mutants to that of the known catalytically inactive D116A integrase (class I mutant, Fig. 6b). Under these conditions, viral cDNA production was found to be unaffected in all the viruses, indicating that there was no significant defect in the reverse transcription step, in contrast to the results observed with RT inhibition (AZT treatment). By contrast, the amount of integrated viral DNA detected for the R231G/A/H mutants was reduced by approximately 25, 60 and 80%, respectively, with a characteristic accumulation of 2-LTR circles over time, which is indicative of normal nuclear import of the pre-integration complex. However, the D253H mutant showed a 20–40% increase in the amount of integrated DNA compared with the WT levels. This increase was associated with a decrease in the quantity of 2-LTR circles, indicating that the integration step was more efficient for this mutant, as confirmed by time-course analyses.

According to the biochemical data, one explanation for these replication phenotypes was a change in the functional association between the mutants intasomes and the chromatine/nucleosomes. To further investigate this hypothesis we next analyzed the chromatin structures surrounding the integration loci.

### Effect of IN/H4 mutations on genomic integration sites selection

**Fig. 7:**
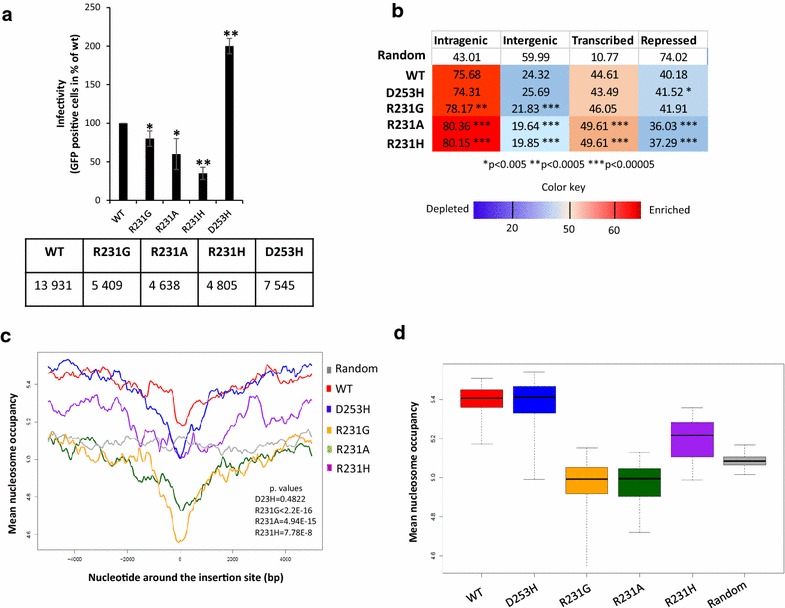
Effect of mutations disturbing the IN/H4 tail interaction on HIV-1 integration site selectivity. K562 cells were transduced with VSV-G pseudotyped lentiviruses encoding either WT IN or the R231A/H/G or D253H IN mutants. Viral replication was quantified based on eGFP fluorescence measured by FACS 48 h post-transduction. The data obtained shown in (**a**) are expressed as the percentage of eGFP-positive cells at a MOI of 1. The number of independent insertion sites analyzed is also reported. Position of human genes and multivariate genome segmentation data were used to count the insertion sites of the WT and the mutant viruses in intra- and intergenic, predicted transcribed and repressed (**b**) regions of the K562 genome [43]. Numbers indicate percentage values of insertion sites per condition. The color code stands for depletion or enrichment in the number of the insertion sites compared to a random expected frequency. The p values were calculated with Fisher’s exact test between the values of WT and the mutants,*p < 0.05 and **p < 0.005. The nucleosome density signal maps were generated from the results of mononucleosome core DNA sequencing using micrococcal nuclease digestion (MNase-seq, [23]) performed on chromatin of K562 cells. Nucleosome occupancy scores in windows of ± 5 kb around the insertion sites is shown for WT and mutant viruses shows the mean nucleosome coverage of the nucleotides around the insertion sites within 10 kb windows (**c**). The gray line depicts the mean nucleosome coverage of nucleotides around a genomic-wide set of random loci. The overall mean nucleosome occupancy values for the ± 5 kb windows around the insertion sites is shown in (**d**). The *y* axes show the average nucleosome occupancy scores around the insertion loci of the WT and the mutant viruses in 4 kb windows around the integration sites in K562 cells. The nucleosome occupancy values measured for random control is reported as a grey line. The p values were calculated by Student’s t-test and are shown as **p < 0.005 to represent the probability of obtaining significant differences compared with WT data

K562 cells were chosen because chromatin features, including histone modifications and nucleosome positions, are well annotated in this cell line. When K562 cells were transduced with lentiviruses carrying the D253H, R231G, R231A and R231H IN versions, we detected a decrease in transduction efficiency of approximately 20, 30 and 60% for the R231G/A/H mutants, respectively, and an increase in efficiency of approximately 40% for the D253H mutant compared with the WT enzyme (Fig. 7a and DNA population analyzes in Additional file 1: Figure S7). Three days post-transduction, the isolated genomic DNA samples of the transduced cells were subjected to integration sites library preparation and high-throughput sequencing.

Between 4638 and 13,931 independent integration sites were obtained and analyzed. In agreement with previous findings [20, 21], analyses using genome-wide histone modification data obtained from ChIP-seq experiments performed on the chromatin of K562 cells showed that the WT insertion sites were underrepresented in heterochromatin (H3K27me3-enriched regions) and highly associated with histone marks characteristic of active transcription and open chromatin, including H3K36me3 (Additional file 1: Figure S8). We detected no significant differences in the distribution of the integration sites of the WT and the mutant INs in chromatin segments with various histone marks. By contrast, the insertion sites of the R231G/A/H mutants were more frequently localized in intragenic regions than those of the WT and D253H vectors (p value = 2.53E−4, 3.68E−11 and 1.68E−10, respectively), and the R231 mutants integrated less frequently in intergenic territories (p value = 1.15E−5, 3.3E−13 and 1.6E−12 for R231G, R231A and R231H, respectively; Fig. 7b). Additionally, the R231A/H integrase substitutions resulted in a significant increase of approximately 5% in the representations of the integrants in transcribed regions compared with those of the WT and D253H versions (p value = 3.91E−9 and 1.67E−8, respectively). Concordantly, integration sites of the R231A/H mutants were less frequently found in repressed genomic territories (p value = 1.72E−20 and 3.51E−15, respectively). In these analyses, the R231G mutant presented an intermediate state, as its preference for intragenic regions and transcribed genes was also affected, but to a lesser extent. Interestingly, the D253H mutant exhibited a trend opposite to that of the R231G/A/H mutants and showed a decreased preference for highly transcribed genes. In summary, we found that the R231 mutants have a stronger bias toward actively transcribed chromatin segments than the WT virus. Since the level of transcription is positively correlated with chromatin accessibility [22], we next studied the nucleosome content of the chromatin neighboring the insertion sites.

The nucleosome occupancy of the chromatin around the insertion loci was analyzed using the results of mononucleosome core DNA sequencing (MNase-seq [23]) performed on chromatin from K562 cells [22]. Similar to previous results [6, 8], measuring nucleosome occupancy in windows of ± 5 kb around the insertion sites showed that insertions of the WT vectors occurred in nucleosome-rich chromatin and that this preference declined toward the immediate insertion locus (Fig. 7c). We also found a lower mean nucleosome occupancy in the chromatin region around the R231G/A/R IN insertions sites with regards to the chromatin region surrounding the WT insertions (Wilcoxon test, p_R231G_ < 2.2E−16, p_R231A_ = 4.94E−15, p_R231H_ = 7.78E−8; Fig. 7c, d). These results suggest that the above vectors carrying IN/H4-disrupting mutations are less biased toward nucleosome-rich target DNA.

Since recent data suggest that residues in the HIV-1 CTD are involved in target DNA binding and recognition [7, 16, 24], we analyzed the nucleotide composition of the integration sites of the mutants. No major changes in the known weak consensus sequence of target site nucleotides typical of the WT IN were detected (Additional file 1: Figure S9). These findings, together with the results of the integration catalysis and DNA binding assays in vitro, argue against the possibility that the altered IN/target DNA interaction is responsible for the changes in the insertion site patterns of the mutants.

Altogether, our findings suggest that mutations disrupting the IN/H4 interaction may decrease the ability of the mutated INto bind and functionally integrate within nucleosomes. This would explain the shift of insertion patterns toward more accessible, dynamic and nucleosome-sparse chromatin regions.

## Discussion

Using multiple complementary approaches, we demonstrated that the presence of histone tails is required for efficient HIV-1 integration into nucleosomes. Additionally, we report here that HIV-1 IN binds histone amino-terminal tails, with a significant preference for the H4 tail. This interaction was shown to be required for efficient interaction with nucleosomes and optimal integration in vitro. Docking calculations, mutagenesis studies and binding analyses enabled us to identify several amino acid positions in the CTD of HIV-1 IN, more precisely in its V-groove, that modulate the interaction between IN and the histone tail. Analysis of the nucleosome-binding properties of the selected mutants and their capability to integrate into nucleosomes showed strong correlations between their ability to bind to the H4 tail and to nucleosomes and their ability to catalyze efficient integration into nucleosomes.

Functional analyses showed that mutations preventing the IN/H4 association also reduced viral infectivity and partly impaired the integration process. A simplest explanation for this phenotype is a deficiency in the interaction between IN and a cellular cofactor. Because all of the mutated enzymes in this study were able to interact with LEDGF/p75 (data not shown), we propose that the loss of the interaction between IN and the histone tails, leading to a loss of interaction with the nucleosome, was directly responsible for the observed integration deficiency. Importantly, the LEDGF/p75 IN cofactor did not affect IN/H4 binding or its effect on MN association and integration. This indicates that the IN/LEDGF and IN/H4 interactions may occur simultaneously, which further suggests the physiological role of this histone interaction. This is also supported by the cellular data indicating that mutations preventing the IN/H4 interaction redirect integration into genes and more dynamic regions of the chromatin. Recent studies have also reported mutations in the CTD that redirect integration. Notably, the R231G polymorphism showed more pronounced integration into GeneSeq genes but in less gene-dense and transcribed regions of the host chromatin [24]. While the redirection of this mutant into genes appears to be consistent with our data, the difference in the preference for less transcribed regions could result from differences between our and the published experimental conditions.

Interestingly, the phenotype reported in our work is reminiscent of that observed for PFV IN, which was recently reported to bind to nucleosomes via the direct interaction of IN with histones, namely, the H2A/H2B dimer surface [10]. Indeed, in both cases, PFV and HIV-1 mutants exhibiting impaired binding to MNs also showed impaired integration and an increased preference for transcribed genes and lower nucleosome occupancy regions ([10] and this work, see Fig. 7). Consequently, these data support the hypothesis that the direct binding of retroviral INto human histones contributes to optimal integration. Retroviral intasomes may have developed various histone-binding mechanisms involving different intasome organizations.

Although several amino acid positions that modulate the HIV-1 IN/H4 interaction, including Y227, D229, R231, K236 and D253, have been identified, the putative histone-binding site has yet to be fully mapped using structural approaches. Indeed, although the mutations introduced in these positions clearly affect the association between IN and histone H4, we cannot conclude at this stage whether these positions are indirectly or directly involved in the interaction. Furthermore, the CTD has also been reported to bind target DNA [7] 33) and reverse transcriptase [17–19], making it difficult to discriminate between these pleiotropic functions and histone binding. Interestingly, the analysis of the cryoEM structure of the HIV-1 STC intasome [16] indicated that the histone tail binding site is accessible in the CTDs of all assembled IN protomers (Additional file 1: Figure S10). The CTDs of the two inner protomers contact the host DNA and are the best candidates for histone tail binding. This observation remains to be verified for the two synaptic CTDs of the lentiviral maedi-visna virus (MMV) STC intasome, whose hexadecameric 4.9 Å resolution cryoEM structure reflects a plausible higher macromolecular assembly for HIV-1 IN [25]. Additionally, these recent structural data also indicate that lentiviral integration is mediated by supramolecular complexes involving a hexadecamer of IN [16, 25]. Thus, these structures show that (1) a CTD within the catalytic protomers can interact with both target DNA and the H4 tail and (2) although some CTDs of the intasome are clearly engaged with target DNA, other CTDs from other non-catalytic protomers may be available for additional protein–protein contacts. For similar reasons, it remains difficult to discriminate between the effect of R231 mutations on target DNA binding, as previously reported [7, 24], and on histone binding as reported here. However, the effect of R231 mutations on nucleotide preferences within the target site has been shown to be considerably lower than that reported for analogous PFV mutations ([16] and our own data (Additional file 1: Figure S6)). This phenotype is better explained by the recently reported structure showing a weaker interaction between the R231 HIV-1 IN residue and target DNA compared with the homologous R229 residue of PFV IN [10, 16]. This is also confirmed by the results of our integration assays and DNA binding experiments reported in Additional file 1: Figures S5 and S6 showing that the catalytic properties of these R231 mutants are not significantly affected. Furthermore, using DNA MCs mimicking the nucleosomal DNA curvature in the absence of histones, we recently showed that mutations in the CTD residues involved in target DNA binding and recognition do not significantly affect their preference for specific DNA curvatures found at the surface of the nucleosome [11]. These data suggest that the change in target nucleosomal DNA selectivity previously observed in vivo [24] likely does not solely result from a loss of target DNA structure recognition but also results from a possible additional interaction with other histone-like components, as reported in our work.

Our data provide also an explanation for the inhibition of HIV-1 integration in dense chromatin templates as previously reported [6, 8]. Indeed, in these polynucleosome templates, the H4 tail is known to interact with neighboring nucleosomes, and access to the tail can be modulated by several processes, such as local chromatin remodeling [26–28]. Interestingly, the integration-refractory property of dense chromatin can be overcome by such remodeling activity (6, 8). These data suggest that local nucleosome remodeling could be required for efficient integration by allowing additional protein/protein interactions between the incoming intasome and the nucleosome, such as the interactions between IN and histones reported herein. Moreover, we have recently shown that local remodeling by the FACT histone chaperone complex allows HIV-1 integration into poly-nucleosomes by generating partially dissociated nucleosomes which fully supports this hypothesis [9]. One direct effect of the chromatin remodeling by FACT would be thus to make accessible the H4 tails for interaction with the incoming intasomes.

Interestingly, the higher impact observed on in vitro integration when using tail less nucleosomes in comparison to H4 TL constructs suggests that several tails may act in synergy to modulate HIV-1 integration. Further structural determination of the intasome/nucleosome contacts by crystallography or cryo-electron microscopy, will be required to fully depict the role of each histone tails as well as histone core in the integration modulation in the context of the functional intasome/nucleosome complex.

## Conclusion

The HIV-1 IN/H4 interaction reported in our work constitutes a new host/pathogen interaction important for the functional association between the incoming intasomes and the targeted nucleosome. Additional cellular processes and additional cellular protein factors, such as the recently discovered CPSF6 protein [39], participate also in regulating this multi-factorial mechanism. Consequently, optimal retroviral integration would result from an equilibrium being reached among efficient chromatin targeting, nucleosome anchoring and recognition of local DNA features. In this complex process, the interaction between IN and the H4 histone tail reported here could be an additional important determinant and, thus, constitute a potential novel therapeutic target.

## Methods

### Proteins, peptides and antibodies

Wild type (WT), mutated full-length and His-tagged truncated HIV-1 INs were purified as previously reported [6, 29]. GST-tagged HIV-1 IN CTD (220–288 amino-acids) was expressed in *Escherichia coli* BL21 cells (DE3) [29]. LEDGF/p75 and IN·LEDGF complex were purified as following the previously reported protocol [30, 31]. Polyclonal anti-HIV-1 IN antibodies were purchased from Bioproducts MD (Middletown, MD, USA). Antibodies directed against histones H3 (ab70550) and H4 (pAb61521 clone MABI 0400) were purchased from Abcam and Active Motif (Carlsbad, CA, USA) respectively. Recombinant mononucleosome assembled on 601 sequence biotinylated in 5′ and the naked corresponding sequence were purchased from TEBU-Bio or were home-made using typical salt dialysis protocole described in [6, 8]. We used either native human histone octamers or tailless octamers purified in the Protein Expression and Purification Facility (PEPF) from the Department of Biochemistry and Molecular Biology, Colorado State University. The quality of the assembly was checked on gel shift in 0.8% agarose gel and protein content analysis on SDS-PAGE (see Additional file 1: Figure S1). Biotinylated peptides were purchased from Eurogentech (Angers, France).

### In vitro integration assays

Concerted integration assays were performed as previously reported [6] using recombinant purified IN or IN•LEDGF/p75 complex (200 nM in IN monomers). IN/viral DNA complex were preassembled using previously optimized conditions [6, 32] and 10 ng of donor DNA containing the U5 viral ends (see description of the different donors in Additional file 1: Figure S11). Preassembled complexes were then incubated with 50 ng of pBSK-derived p481 plasmid DNA in 20 mM HEPES pH7, 15% DMSO, 8% PEG, 10 mM MgCl2, 20 µM ZnCl2, 100 mM NaCl, 10 mM DTT final concentration.

After the reaction, the resultant integration products were deproteinized by Proteinase K treatment and phenol/chloroform/isoamyl alcohol (25/24/1 v/v/v) treatment before loading onto a 1% agarose gel. The gel was then dried and submitted to autoradiography. The bands corresponding to free substrate (S), donor/donor, linear FSI (FSI) and circular HSI + FSI (HSI + FSI) products were quantified. The circular FSI products were specifically quantified by cloning them into bacteria and determining the numbers of ampicillin-, kanamycin- and tetracycline-resistant clones as percentages of the integration reaction control, which was performed using the WT enzyme. Integration assays using recombinant 601 mononucleosomes or naked 601 DNA fragments were performed using the same procedure, except that a shorter viral DNA fragment corresponding to the 42 final base pairs of the HIV-1 U5 viral ends was used (see sequence in Additional file 1: Figure S11) and the concentration of IN was increased to 400 nM. Either 250 ng of MN or 125 ng of acceptor DNA were used. Acceptor substrates were immobilized on streptavidin-coupled beads before reaction and the reaction products were deproteinized as described above and the integration was quantified by counting the remaining radioactivity bound to magnetized beads.

### Docking calculations

In all docking experiments, the fragment corresponding to residues A210-A270 from the HIV-1 IN catalytic core and the CTD crystal structure (PDB entry 1EX4) [15] was used as a protein receptor. For the ligand, we used the crystal structure of the H4K20me1 pentapeptide from the human MSL3 chromodomain complex (PDB entry 3OA6) [33]. The receptor and ligand structures were prepared for docking with AutoDockTools 1.5.6 [34]. Polar hydrogen atoms were added, non-polar hydrogens were merged, and Gasteiger partial atomic charges were computed. All possible rotatable bonds were subsequently assigned for the H4K20me1 ligand molecule. In the first set of experiments, a blind docking was performed on the entire surface of the receptor, which was treated as rigid, using the programs AutoDock 4.2.6 [34] and AutoDockVina 1.1.2 [35]. The combined docking results from these two methods enabled us to determine a unique consensus binding area. Second, experiments focusing on this area were conducted to predict the residues that may be involved in the binding of the ligand. To this end, a set of 14 residue side-chains surrounding the predicted binding area was treated as flexible. AutoGrid was used to produce grid maps that were properly centered to encompass the area of interest, with a grid box size of 76 × 84 × 98 points and a grid spacing value of 0.264 Å. AutoDock performed a total of 1000 independent runs with step sizes of 0.2 Å for translations and 5 Å for torsions. The Lamarckian Genetic Algorithm was used with a population size of 150 individuals, the maximum number of energy evaluations set to 10,000,000, the maximum number of generations set to 27,000, the maximum number of top individuals that automatically survived set to 1, and mutation and crossover rates of 0.02 and 0.8, respectively. The final cluster analysis of all docked conformations was achieved with a cluster tolerance of 3.5Å. Finally, the top-ranked docking solutions were analyzed with AutoDockTools.

### Pull-down experiments

Recombinant purified WT, mutant HIV-1 INs or IN•LEDGF/p75 complex (10 pmol of IN monomers) were incubated with either native recombinant W601 mononucleosomes, tailless MNs (250 ng, i.e., 125 ng DNA), or the naked 601 DNA sequence (125 ng) in 10 µl interaction buffer (50 mM HEPES, pH7.5; 1 µg/ml BSA; 1 mM DTT; 0.1% Tween 20;10% glycerol; and 50–240 mM NaCl) for 20 min on ice and then for 30 min at room temperature. A 12.5 µl aliquot of DynabeadsMyOne Streptavidin T1 (Invitrogen, ref. 65601) was then added to a total volume of 300 µl interaction buffer and incubated at room temperature for 1 h under rotation. The beads were washed three times with 300 µl interaction buffer, and the precipitated products were resuspended in 10 µl of Laemmli buffer, after which they were separated on a 12% gel via SDS-PAGE. Interacting proteins were detected by western blot analysis using anti-HIV-1 IN and anti-histone antibodies. Nucleosomal DNA was detected using a 1% agarose gel stained with SYBR^®^ Safe. 140–240 mM NaCl conditions were chosen for analyzes since salt concentrations lower than 140 mM led to unspecific binding of HIV-1 INto the beads masking its interaction with nucleosomes.

### FAR dot blot experiments

One µl of HIV-1 IN solution (1–10 pmol) was spotted onto a nitrocellulose membrane and dried for 1 h at room temperature. The membrane was then saturated for 3 h at room temperature with 5 ml of 1% BSA in PBS. After two washes, the membrane was incubated with 1 µM of the requisite peptide in 4 ml of PBS for 1 h at 37 °C. After two washes with PBS, the membrane was incubated with ExtrAvidin coupled to horseradish peroxidase (Sigma ref. E2886 1/4000) in 4 ml of 0.3% BSA in PBS for 1 h at room temperature. The interactions were detected by ECL using a LAS4000 device. The far dot blots were run three to ten times and the intensity of each spot was quantified using ImageJ software.

### Transduction of human cells with lentiviral vectors

HEK-293T (Human Embryonic Kidney 293 cells, laboratory cell line) were transduced as previously described [36]. An optimized multiplicity of infection (MOI) of 1 was used, which resulted in 25–35% of the cells containing one copy of proviral DNA as determined before. Fluorescence was quantified 48 h post-transduction by counting 10,000 cells on a FACSCalibur flow cytometer (Becton–Dickinson, San Jose, CA, USA). HIV-1 DNA species were quantified at 24, 48 and 72 h post-transduction as previously described [37]. The total and integrated HIV-1 DNA levels were determined as copy numbers per 10^6^ cells. Integrated cDNA and 2-LTR circles were expressed as a percentage of the total viral DNA.

### Integration site library preparation

To remove any non-integrated viral DNA (and one-, or two-LTR circles) per condition, 5 µg genomic DNA (gDNA) samples isolated from K562 (human immortalized myelogenous leukemia cell line purchased from ATCC company) 72 h post-transfection were subjected to 0.6% agarose gel electrophoresis and high-molecular gDNA was isolated from the gel using the Zymoclean™ Large Fragment DNA Recovery Kit, (Zymo Research). The eluents were sonicated to an average of 600 bp-long fragments in screw-cap cuvettes with the Covaris M220 ultrasonicator with the following settings: peak power: 50.0, duty factor: 20, cycle/burst: 200, duration: 28 s. After bead purification the DNA was end-repaired and 5′-phosphorylated with the NEBNext End Repair Module (New England Biolabs, (NEB)). The DNA was prepared for ligation with NEBNextdA-Tailing Module, (NEB) and eluted after bead purification in 10 µl water. Ligation with double-stranded linkers (see Additional file 1: Figure S10) was performed in 15 µl for 15 min at room temperature using the Blunt/TA Ligase Master Mix (NEB). After purification with 0.8 volumes of AMPure XP beads (Beckman Coulter), the ligated DNA was eluted in 20 µl of 10 mM Tris/HCl, pH 8.0 and the whole DNA solution was used for multiple PCR reactions to amplify the virus-gDNA junctions with the primers SIN-HIV1 and linker primer using NEBNext High-Fidelity 2× PCR Master Mix (NEB) with the following cycling conditions: 98 °C 30 s; 20 cycles of: 98 °C 10 s, 68 °C 30 s, 72 °C 30 s; 10 cycles of: 98 °C 10 s, ramp to 63 °C 1 °C/s 30 s, 72 °C 30 s; 72 °C 3 min. The PCR products were isolated using 1 volume of AMPure XP beads (Beckman Coulter), eluted in 20 µl of 10 mM Tris/HCl, pH 8.0 and 2 µl of the eluents served as template for 5 parallel PCR reactions with the primers: SIN-HIV-BC-N-Ill and PE-nest ind-N (where N stands for the sequences of Illumina TrueSeq indexes, or their corresponding reverse complement sequences) using the following cycling conditions: 98 °C 30 s; 20 cycles of: 98 °C 10 s, 67 °C 30 s, 72 °C 30 s; 72 °C 3 min. The 200–500 bp size range of the indexed libraries were agarose gel-isolated and mixed equimolarly for 100 base, single-end Illumina sequencing on a HiSeq 2000 instrument using 40% PhiX DNA spike-in at Genewiz, USA.

### Analysis of sequencing data

The raw reads starting with condition-specific indexes were grouped and filtered for the presence of the virus-specific nested primer followed by LTR sequences at the tip of the LTR. The rest of the reads were quality-trimmed as soon as 2 out of 5 bases had quality scores less than a Phred score of 20. We used *bowtie* [38] with the TAPDANCE tool [39] to map the reads to the hg19 human genome assembly in cycles with decreasing read length of 60, 55, 50, 45, 40, 35 allowing 3, 3, 3, 2, 1, 0 mismatches, respectively, with the following bowtie parameters in the mapping cycles: [–*quiet* -*a* -*v* < *nu. mismatches allowed* > -*m 1* –*suppress 5,6,7* –*f*]. Any insertion site was considered valid if there were at least 5 independent reads supporting it. All read pre-processing and follow-up analyses were done in R (R Development Core Team (2008). R: A language and environment for statistical computing. R Foundation for Statistical Computing, Vienna, Austria. ISBN 3-900051-07-0, URL http://www.R-project.org).

### Analysis of insertion sites in chromatin features

Nucleosome occupancy signal datasets for K562 cells were obtained from ENCODE [22]. Genomic coordinates with an associated nucleosome occupancy density signal value greater than zero were used to calculate occupancy matrixes and to plot nucleosome densities with the *genomation* R package [40]. BEDTools [41] and *genomation* were used to analyze the representation of ISs in histone mark distributions [42] and in chromatin state segment datasets making use of a consensus merge of the segmentations produced by the ChromHMM and Segway software [43]. We applied the Wilcoxon test on the row-sums of the score matrixes generated from nucleosome occupancy datasets to check for any statistical difference between the conditions. Fisher’s exact test was used to calculate statistical significance between the representations of ISs of the WT and the integrase mutant viruses within methylated histone ChIP-seq peaks.

## Additional files

**Additional file 1: Figure S1.** Structure of the native and tailless mononucleosomes used in the work. The globular structure of the nucleosomes was analyzed by loading 250 ng of native or tailless MN on 0.8% native agarose gel run 4 h at 50 V and 4 °C then stained with SYBR^®^Safe 20 min. Assembled MNs migrate between 600 and 700 bp and the naked 601 DNA fragment at 147 bp. **Figure S2.** A. Effect of LEDGF/p75 on HIV-1 integration in vitro. Integration assay was performed as done in Fig. 2 using naked 601 DNA coupled to magnetic beads and increasing concentration of LEDGF in the presence or absence of PEG and DMSO. B. Integration activity catalyzed by IN and IN/LEDGF complex on native or tail less nucleosomes in the absence of PEG and DMSO. Integration assay was performed as done in Fig. 2 using native or tail less nucleosomes coupled to magnetic beads and either IN or IN/LEDGF complex. All values are shown as the mean ± standard deviation (error bars) of three independent sets of experiments. The p values were calculated by Student’s t test and are shown as *p < 0.05 to represent the probability of obtaining significant differences compared with the data obtained with the native MNs control. **Figure S3.** Sequence of the peptide tails used in the work. **Figure S4.** FAR dot-blot analysis of the interactions between HIV-1 IN and peptides derived from histone 4 amino-terminal tails. The associations between IN and unmodified H4, or modified H4 peptides were evaluated using a far dot blot approach as described in the "Methods" section using 1 µl of 2.5 pmol of recombinant IN spotted onto a nitrocellulose membrane and 1 µM of peptides. The far dot blots were run three to ten times and the intensity of each spot was quantified using ImageJ software. The results are reported as the mean of the experiments ± standard deviation. **Figure S5.** In vitro integration activities of wild type and mutant integrases. A concerted integration assay was performed using 200 nM of different enzymes which were purified using a similar procedure, in addition to 10 ng of donor DNA and 50 ng of pBSK-derived p481 plasmid DNA. The reaction products were loaded onto 1% agarose gels and a representative set of experiments is shown in (A). The positions and structures of the donor substrate and the different half-site (HSI), full-site (FSI) and donor/donor integration (d/d) products are shown. Quantification of the total integration is shown in (B) as a percentage of WT activity. The circular FSI products were quantified by cloning them into bacteria and are shown in (C) as the numbers of ampicillin-, kanamycin- and tetracycline-resistant clones as percentages of the integration reaction control performed using the WT enzyme. All values are shown as the mean ± standard deviation (error bars) of at least three independent sets of experiments. The p-values were calculated by Student’s t-test and are shown as *p < 0.05 and **p < 0.005 to represent the probability of obtaining significant differences compared with WT data set at 100%. **Figure S6.** HIV-1 IN and mononucleosome pull-down experiment. Naked 147 bp 601 DNA sequence or MN assembled on this fragment were used (structure of the naked and assembled 601 DNA is reported in the gel shift experiment shown in **Figure S1.** WT IN was efficiently pulled down using a biotinylated naked 601 DNA fragment (left panel) or 601 mononucleosomes assembled on the same DNA (right panel) immobilized on streptavidin beads using 140–240 mM of NaCl (A). Experiments were performed using different mutated enzymes. Each pull-down was run three to six times and the intensity of each band was quantified using ImageJ software. The results obtained with naked DNA are reported as the mean of the experiments ± standard deviation in (B). The p values were calculated by Student’s t-test and are shown as *p < 0.05 and **p < 0.005 to represent the probability of obtaining significant differences compared with the WT data in each condition. **Figure S7.** Time course analysis of the early steps of replication of wild type and mutants viral vectors in K562 cells. K562 cells were transduced with VSV-G pseudotyped lentiviruses encoding either WT IN or the R231A/H/G or D253H IN mutants. The replication steps affected by the mutations were determined by measuring the amounts of the different viral DNA species produced using qPCR. Levels of total viral DNA, integrated DNA and 2-LTR were monitored between 0 and 72 h post-transduction to check for potential defects at the steps of reverse transcription, integration and nuclear import of the preintegration complex. The data are represented as the mean of at least three independent experiments ± standard deviation. The p-values were calculated by Student’s t-test and are shown as *p < 0.05 and **p < 0.005 to represent the probability of obtaining significant differences compared with the WT data. **Figure S8.** Effect of mutations affecting the IN/H4 tail interaction on HIV-1 integration site selectivity. Integration sites of the WT and the mutant viruses were annotated in signal peaks of ChIP-seq experiments for genome-wide histone modifications in K562 cells. Numbers indicate percentage values of insertion sites per condition. The p values were calculated with Fisher’s exact test between the values of WT and the mutants,*p < 0.05 and **p < 0.005. **Figure S9.** Consensus sequences directly neighboring insertion sites of pseudoviral vectors carrying IN/H4 mutations. The target DNA consensus diagrams were generated with the *seqLogo* package in R. The triangles show the insertion sites. The relative height of individual bases at each position is proportional to the frequency of the base at that position. **Figure S10.** Superimposition of the HIV-1 IN CTD-H4K20me1 docking model with the structure of the tetrameric HIV-1 strand transfer complex intasome (PDB entry 5U1C). The model is presented in magenta cartoon representation with the docked H4K20me1 pentapeptide highlighted in green. The CTDs of the two inner protomers contacting the host DNA (colored in gold) are depicted in salmon and cyan. The grey cartoon corresponds to the rest of the HIV-1 strand transfer complex structure. **Figure S11.** Sequence of the viral DNA donors used in concerted integration assays. For concerted integration on MNs the two HIV1_U5 (+) and HIV1_U5 (-) (A) were hybridized and the resulting 42/40 bp hybrid was radiolabeled in 5′ with T4 DNA kinase. For concerted integration on naked DNA plasmid we used the 246 bp DNA fragment shown in (B) generated by PCR on a pUC19 supF. After purification the fragment was radiolabeled in 5′ with T4 DNA kinase. Sequence of oligonucleotides used for integration selectivity analyses (C).

**Additional file 2.** List of insertion sites of all the tested conditions. Raw sequencing reads are available upon request.
