# Supplementary material for: Modulation of the functional association between the HIV-1 intasome and the nucleosome by histone amino-terminal tails
Source: Retrovirology. 2017 Nov 28;14:54. doi: 10.1186/s12977-017-0378-x (PMC5704366; doi:10.1186/s12977-017-0378-x)
Supplement: Supplementary file 1 — Additional file 1: Figure S1. Structure of the native and tailless mononucleosomes used in the work. The globular structure of the nucleosomes was analyzed by loading 250 ng of native or tailless MN on 0.8% native agarose gel run 4 h at 50 V and 4 °C then stained with SYBR®Safe 20 min. Assembled MNs migrate between 600 and 700 bp and the naked 601 DNA fragment at 147 bp. Figure S2. A. Effect of LEDGF/p75 on HIV-1 integration in vitro. Integration assay was performed as done in Fig. 2 using naked 601 DNA coupled to magnetic beads and increasing concentration of LEDGF in the presence or absence of PEG and DMSO. B. Integration activity catalyzed by IN and IN/LEDGF complex on native or tail less nucleosomes in the absence of PEG and DMSO. Integration assay was performed as done in Fig. 2 using native or tail less nucleosomes coupled to magnetic beads and either IN or IN/LEDGF complex. All values are shown as the mean ± standard deviation (error bars) of three independent sets of experiments. The p values were calculated by Student’s t test and are shown as *p < 0.05 to represent the probability of obtaining significant differences compared with the data obtained with the native MNs control. Figure S3. Sequence of the peptide tails used in the work. Figure S4. FAR dot-blot analysis of the interactions between HIV-1 IN and peptides derived from histone 4 amino-terminal tails. The associations between IN and unmodified H4, or modified H4 peptides were evaluated using a far dot blot approach as described in the "Methods" section using 1 µl of 2.5 pmol of recombinant IN spotted onto a nitrocellulose membrane and 1 µM of peptides. The far dot blots were run three to ten times and the intensity of each spot was quantified using ImageJ software. The results are reported as the mean of the experiments ± standard deviation. Figure S5. In vitro integration activities of wild type and mutant integrases. A concerted integration assay was performed using 200 nM of different enzymes w [file 12977_2017_378_MOESM1_ESM.pptx]

## Slide 1
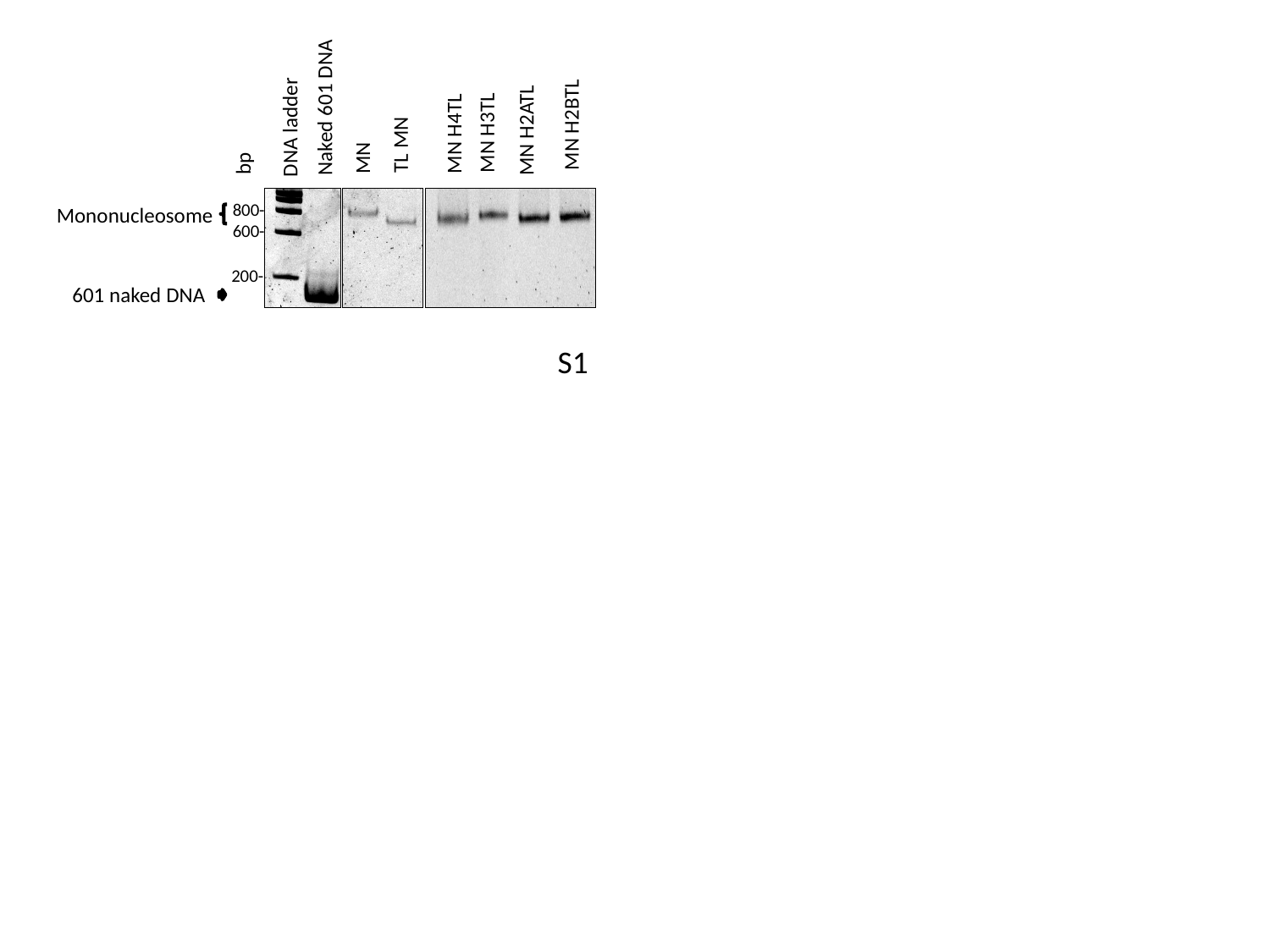

Naked 601 DNA
MN H2BTL
DNA ladder
MN H2ATL
MN H3TL
MN H4TL
TL MN
MN
bp
800-
Mononucleosome
600-
200-
601 naked DNA
S1

## Slide 2
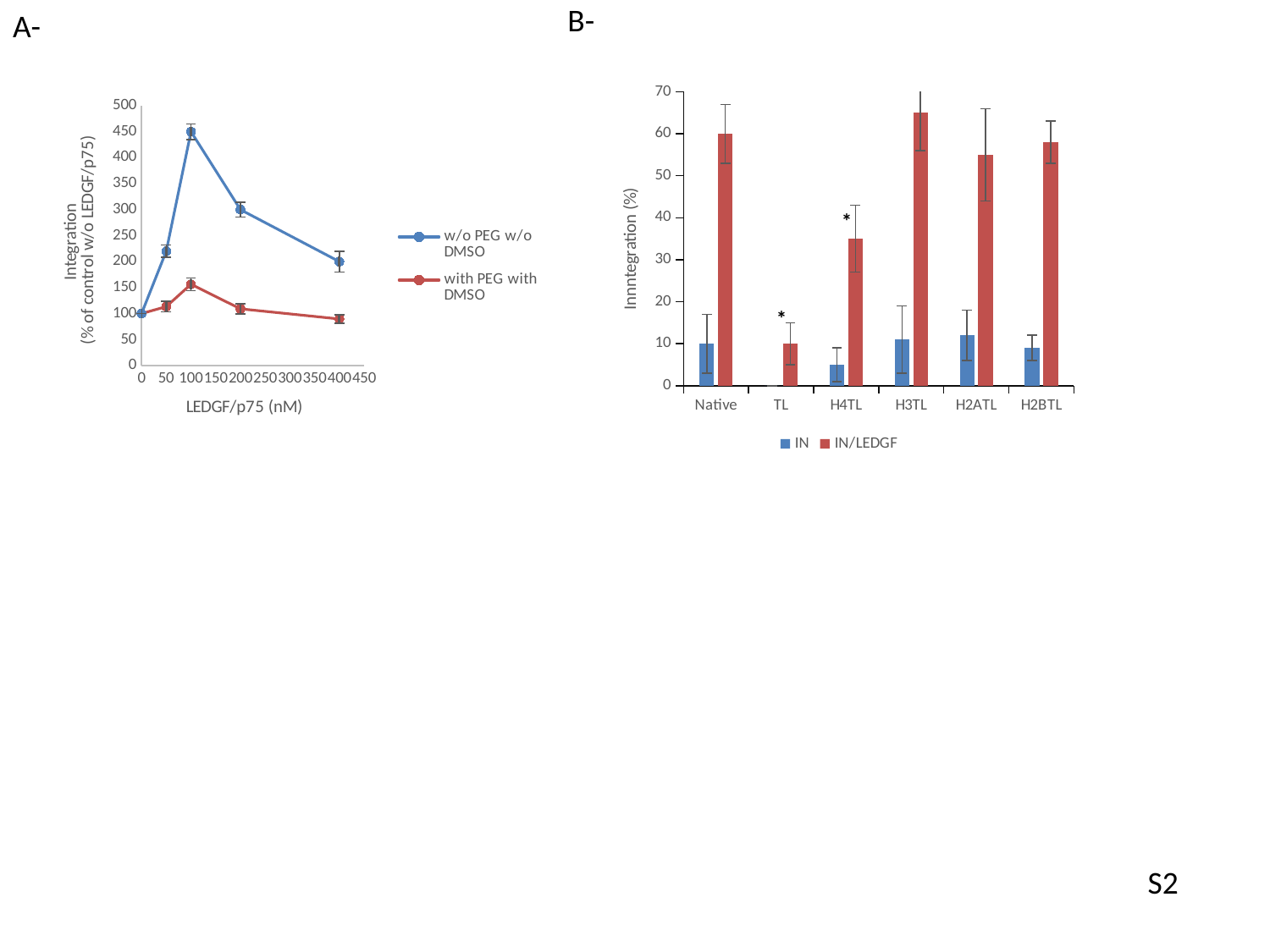

A-
B-
### Chart
| Category | IN | IN/LEDGF |
|---|---|---|
| Native | 10.0 | 60.0 |
| TL | 0.0 | 10.0 |
| H4TL | 5.0 | 35.0 |
| H3TL | 11.0 | 65.0 |
| H2ATL | 12.0 | 55.0 |
| H2BTL | 9.0 | 58.0 |
### Chart
| Category | | |
|---|---|---|*
*
S2

## Slide 3
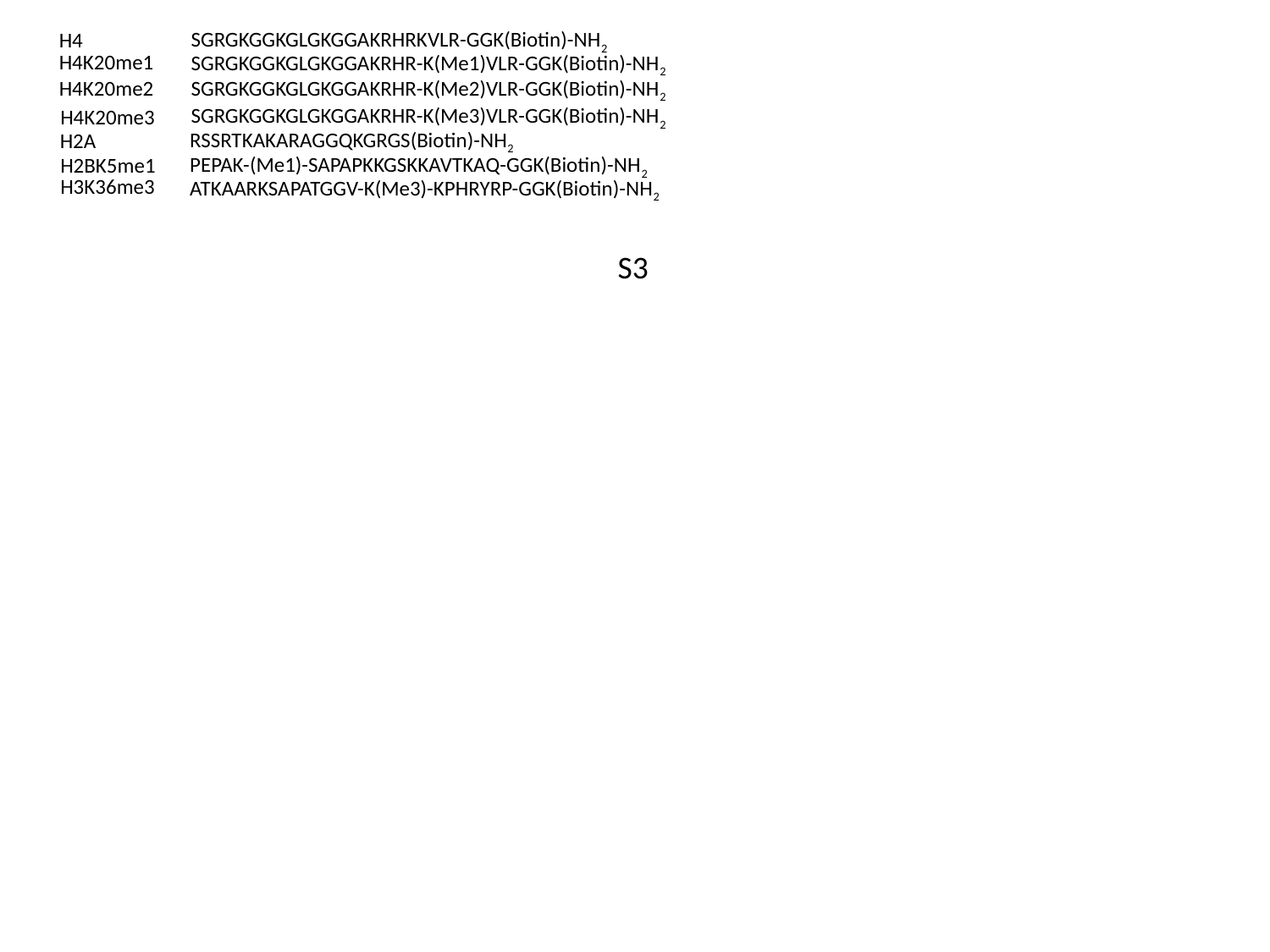

SGRGKGGKGLGKGGAKRHRKVLR-GGK(Biotin)-NH2
H4
H4K20me1
SGRGKGGKGLGKGGAKRHR-K(Me1)VLR-GGK(Biotin)-NH2
SGRGKGGKGLGKGGAKRHR-K(Me2)VLR-GGK(Biotin)-NH2
H4K20me2
SGRGKGGKGLGKGGAKRHR-K(Me3)VLR-GGK(Biotin)-NH2
H4K20me3
RSSRTKAKARAGGQKGRGS(Biotin)-NH2
H2A
PEPAK-(Me1)-SAPAPKKGSKKAVTKAQ-GGK(Biotin)-NH2
H2BK5me1
H3K36me3
ATKAARKSAPATGGV-K(Me3)-KPHRYRP-GGK(Biotin)-NH2
S3

## Slide 4
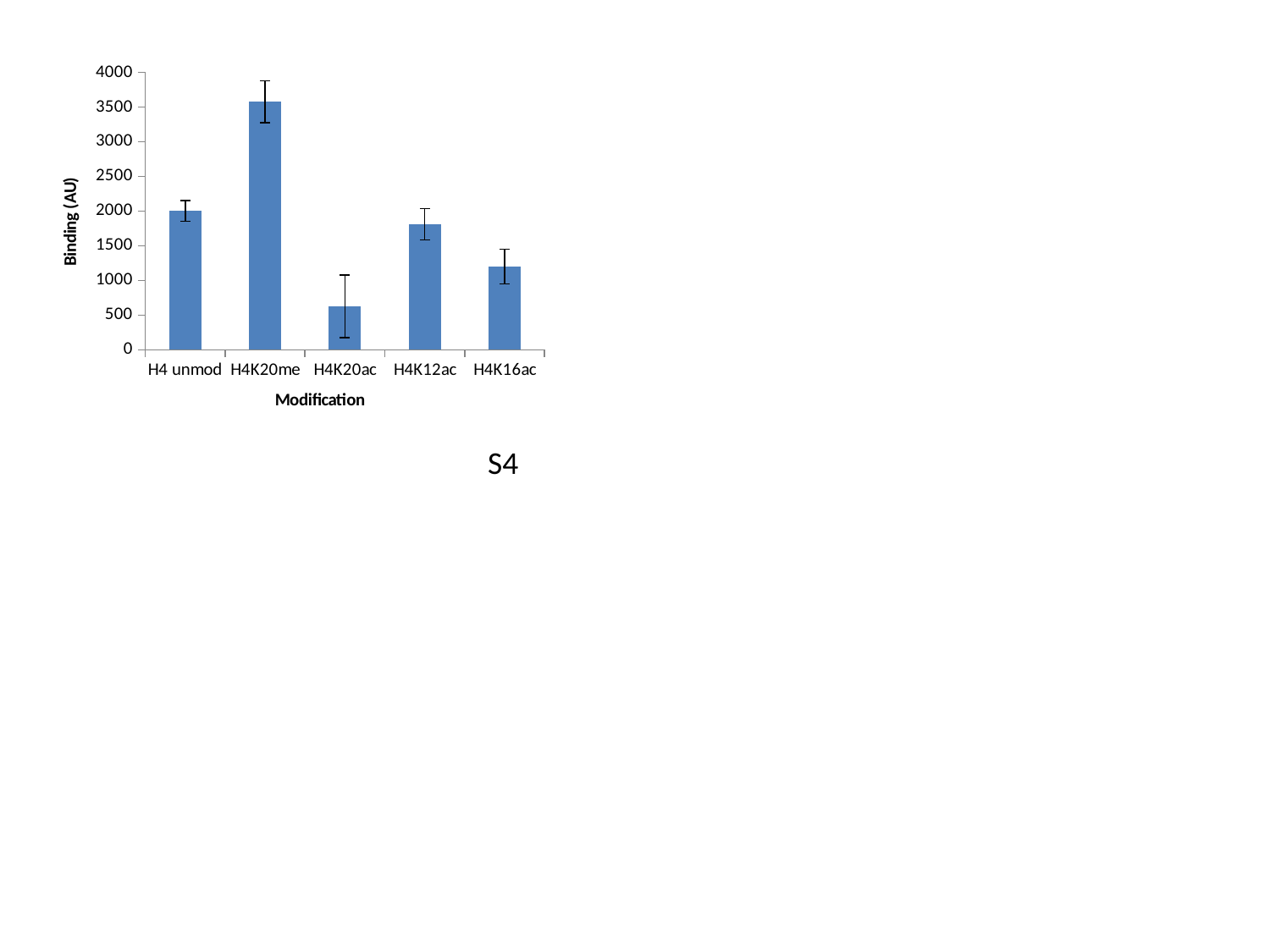

### Chart
| Category | |
|---|---|
| H4 unmod | 2000.0 |
| H4K20me | 3575.04048032688 |
| H4K20ac | 626.3830680425303 |
| H4K12ac | 1808.6722221693203 |
| H4K16ac | 1200.0 |S4

## Slide 5
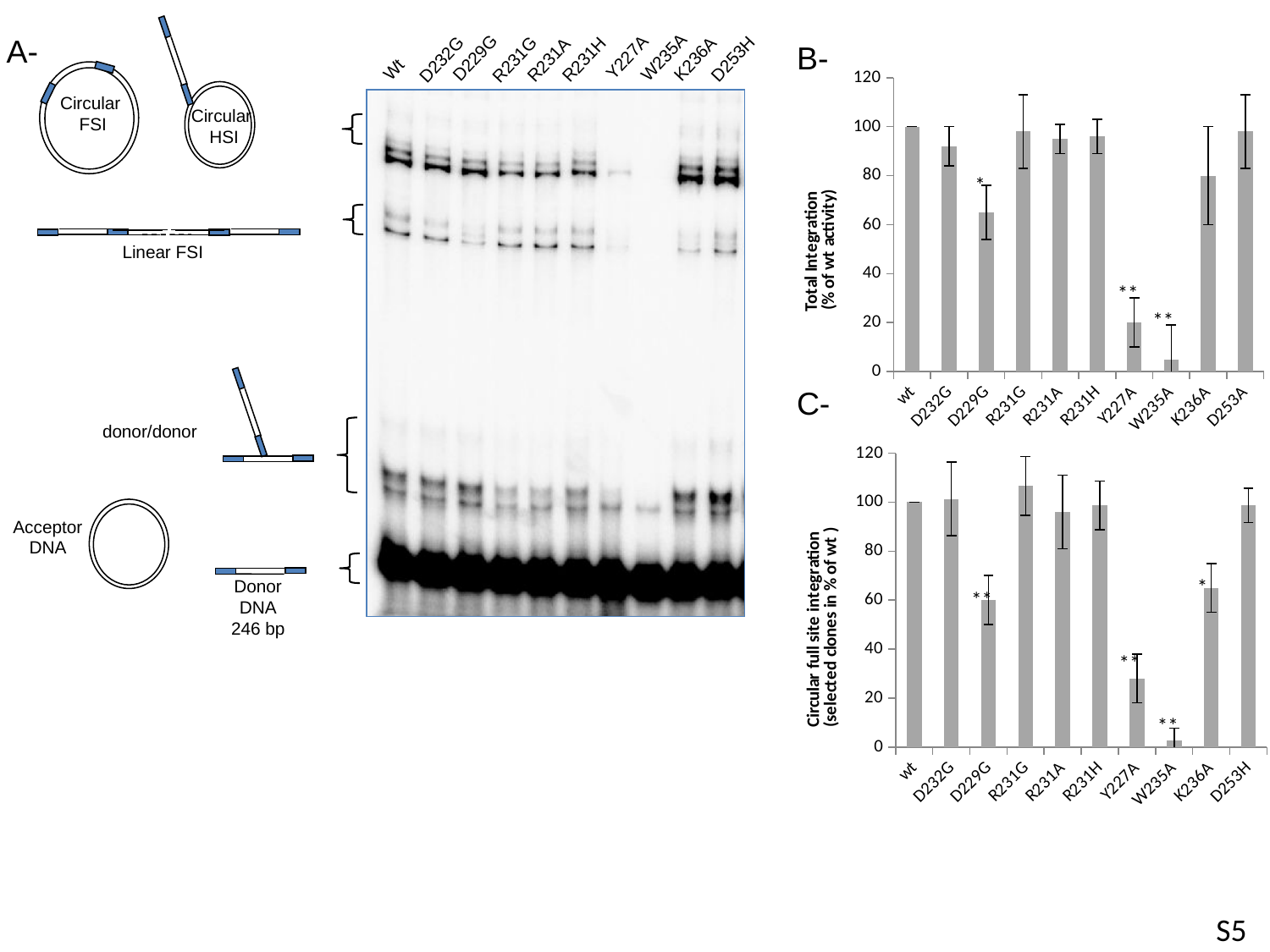

A-
B-
Y227A
W235A
D229G
K236A
D253H
R231H
D232G
R231G
R231A
Wt
### Chart
| Category | |
|---|---|
| wt | 100.0 |
| D232G | 92.0 |
| D229G | 65.0 |
| R231G | 98.0 |
| R231A | 95.0 |
| R231H | 96.0 |
| Y227A | 20.0 |
| W235A | 5.0 |
| K236A | 80.0 |
| D253A | 98.0 |
Circular
FSI
Circular
HSI
*
Linear FSI
**
**
C-
donor/donor
### Chart
| Category | |
|---|---|
| wt | 100.0 |
| D232G | 101.33333333333334 |
| D229G | 60.0 |
| R231G | 106.66666666666667 |
| R231A | 96.0 |
| R231H | 98.66666666666667 |
| Y227A | 28.000000000000004 |
| W235A | 2.666666666666667 |
| K236A | 65.0 |
| D253H | 98.66666666666667 |
Acceptor
DNA
*
Donor
DNA
246 bp
**
**
**
S5

## Slide 6
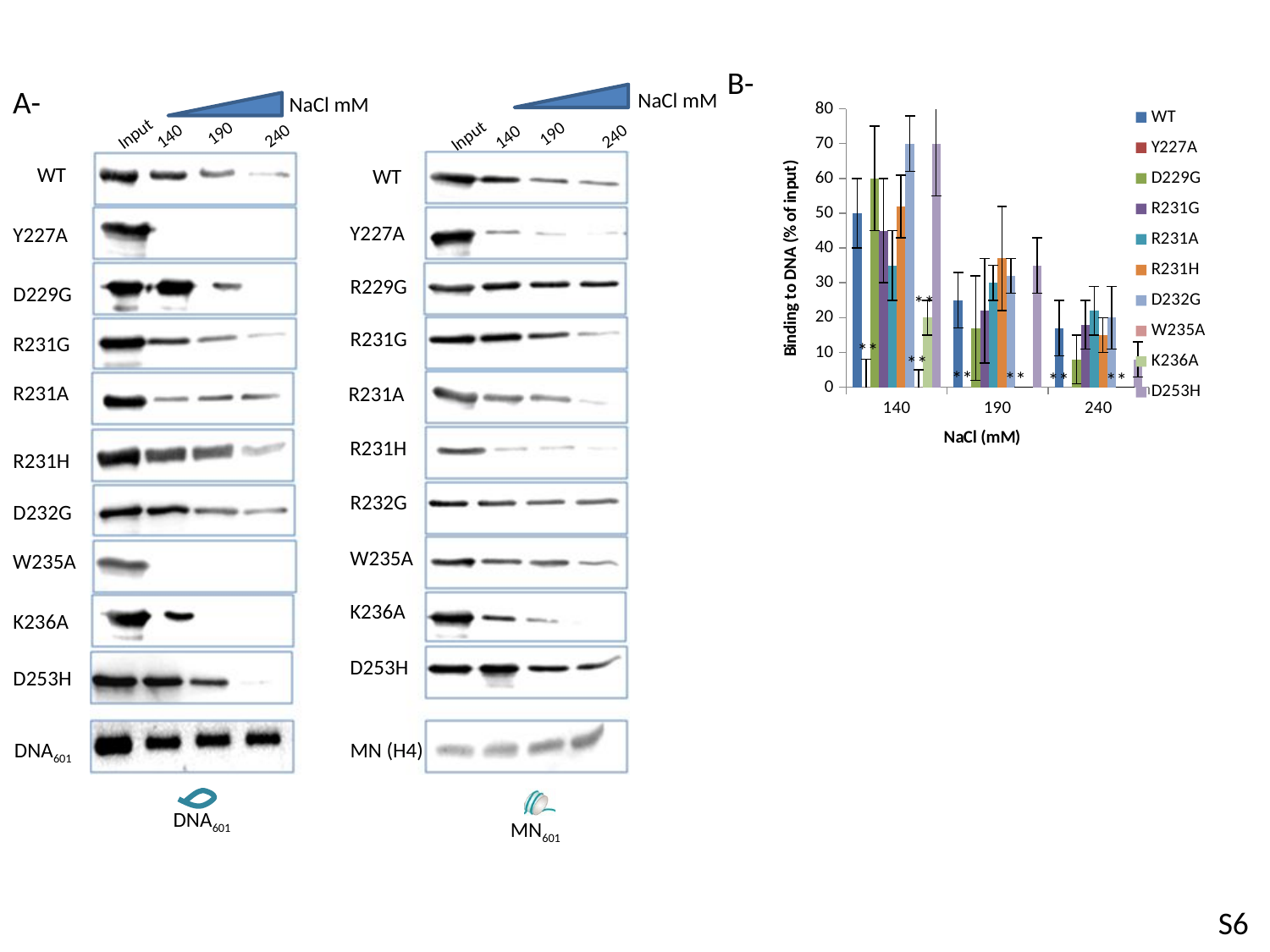

B-
A-
NaCl mM
NaCl mM
[unsupported chart]
190
Input
190
240
Input
240
140
140
WT
WT
Y227A
Y227A
R229G
D229G
**
R231G
R231G
**
**
**
**
**
**
R231A
R231A
R231H
R231H
R232G
D232G
W235A
W235A
K236A
K236A
D253H
D253H
DNA601
MN (H4)
DNA601
MN601
S6

## Slide 7
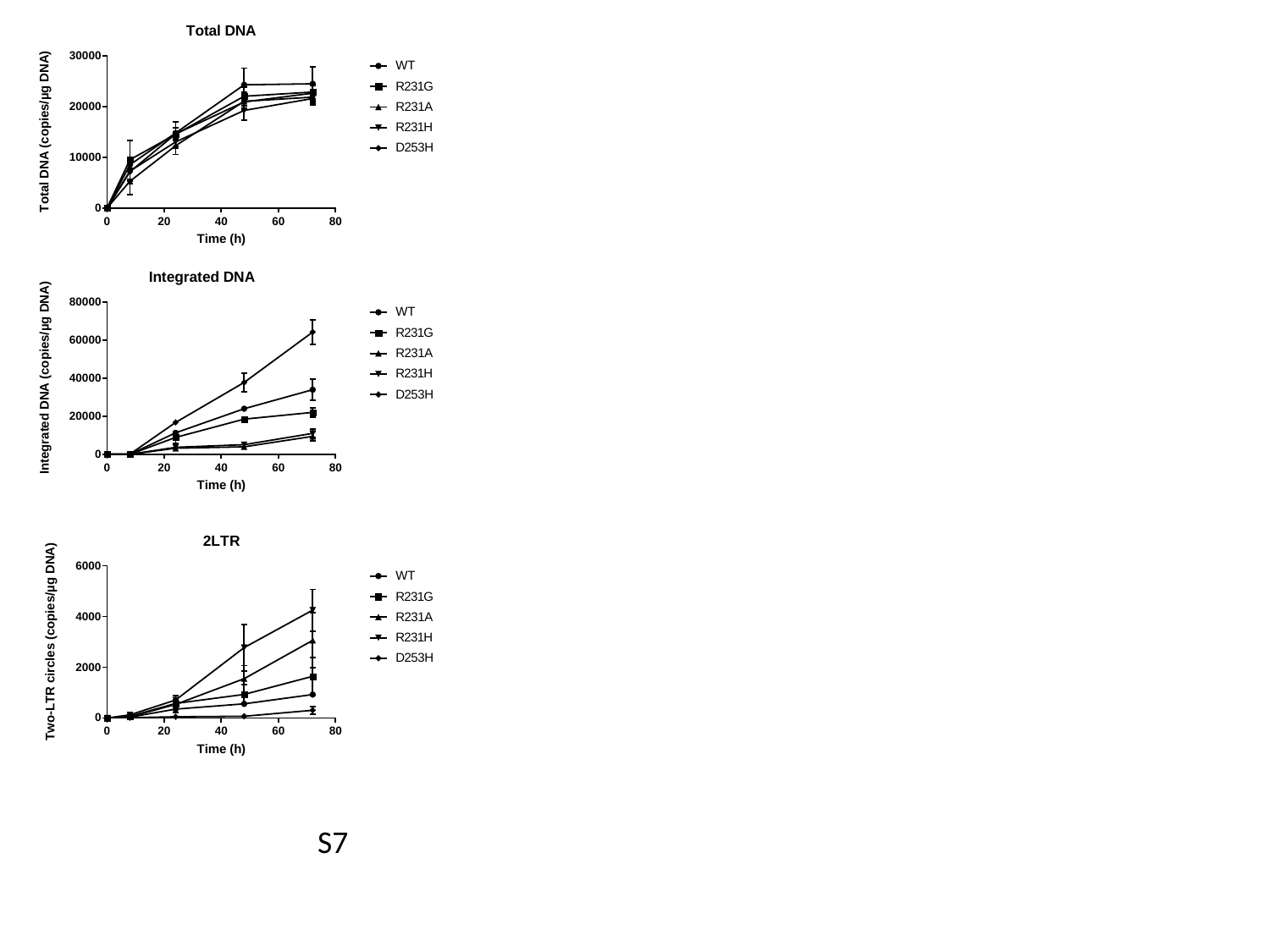

S7

## Slide 8
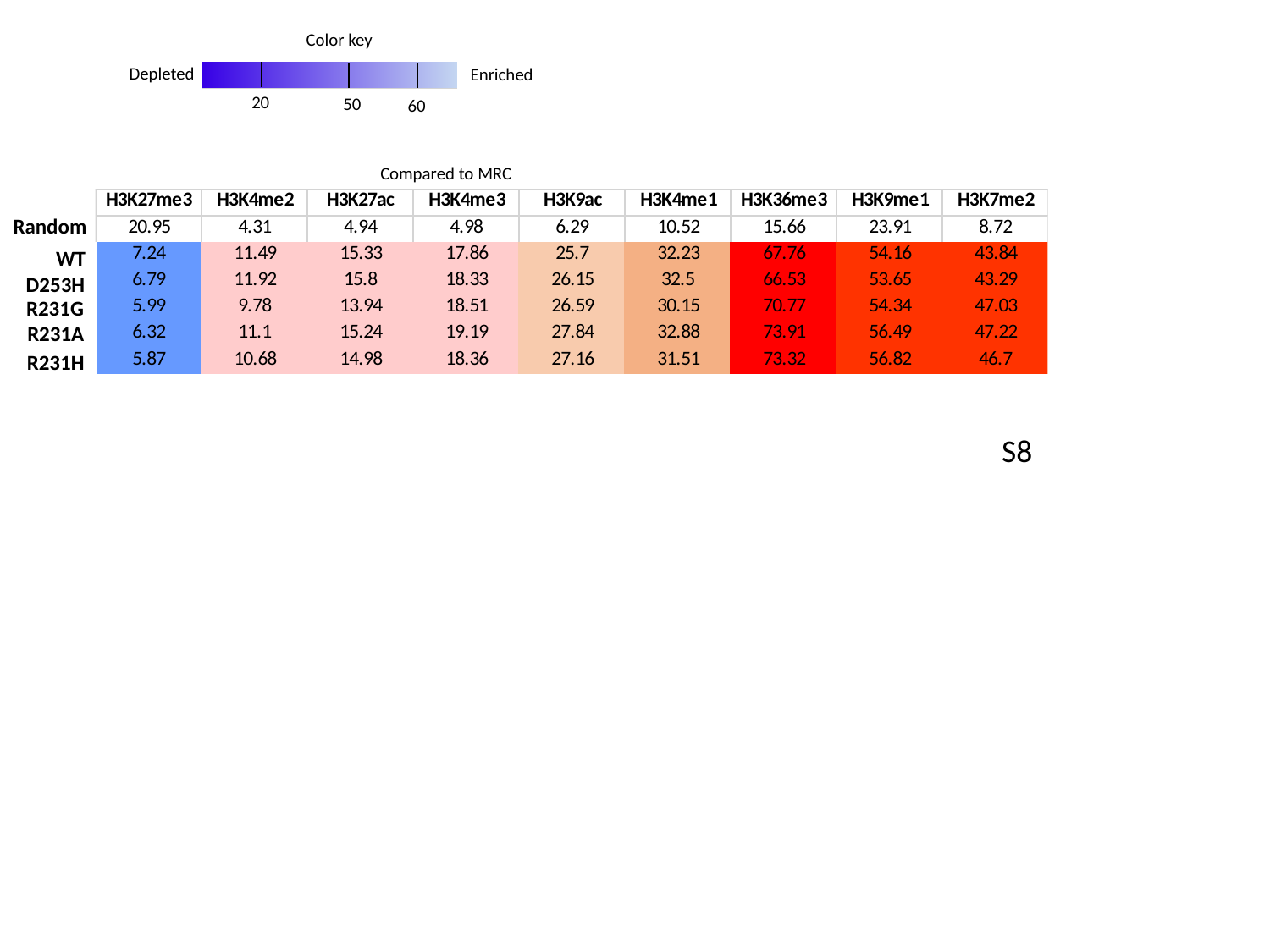

Color key
Depleted
Enriched
20
50
60
Compared to MRC
Random
WT
D253H
R231G
R231A
R231H
S8

## Slide 9
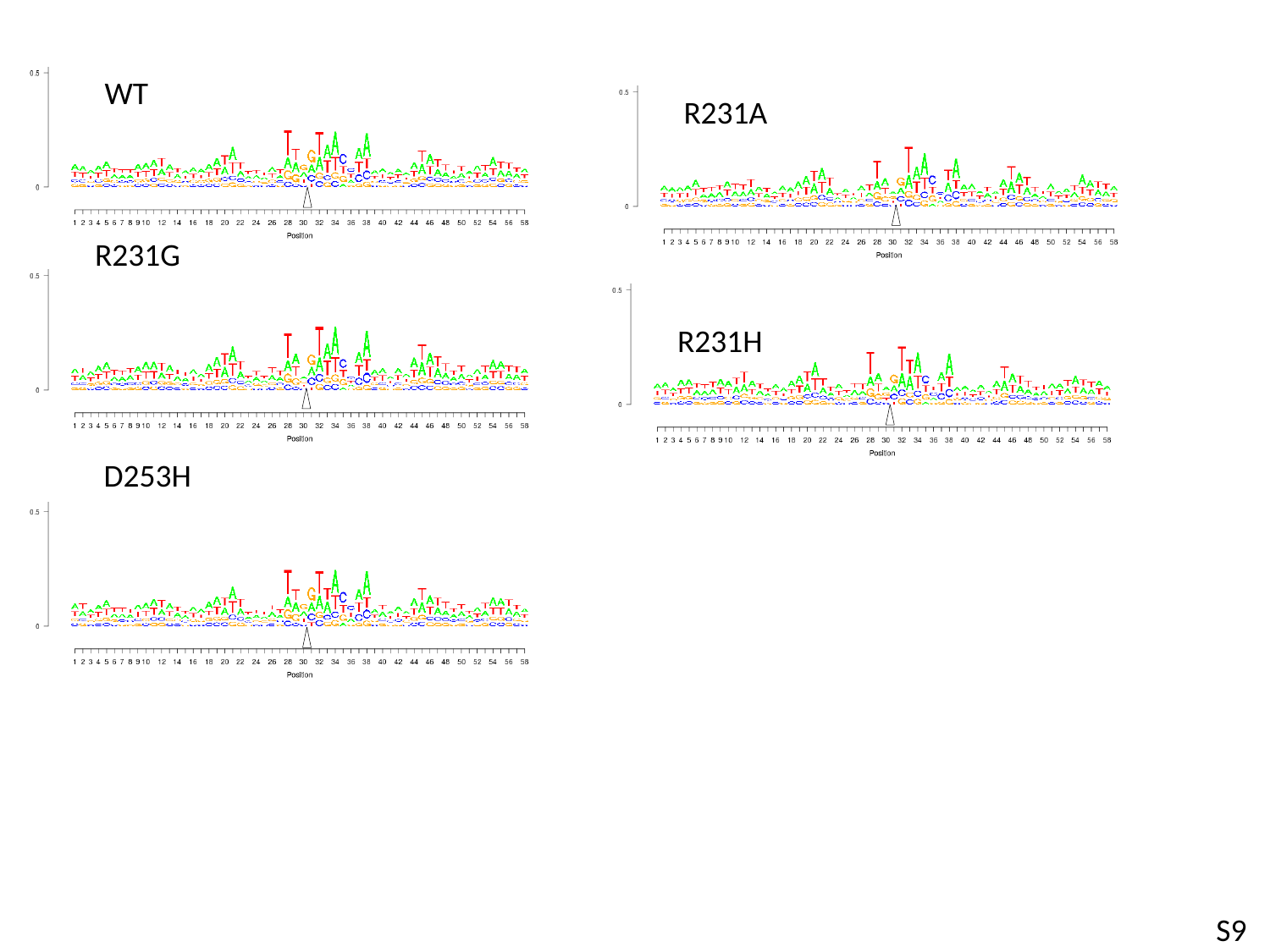

### Chart
| Category |
|---|WT
R231A
R231G
R231H
D253H
S9

## Slide 10
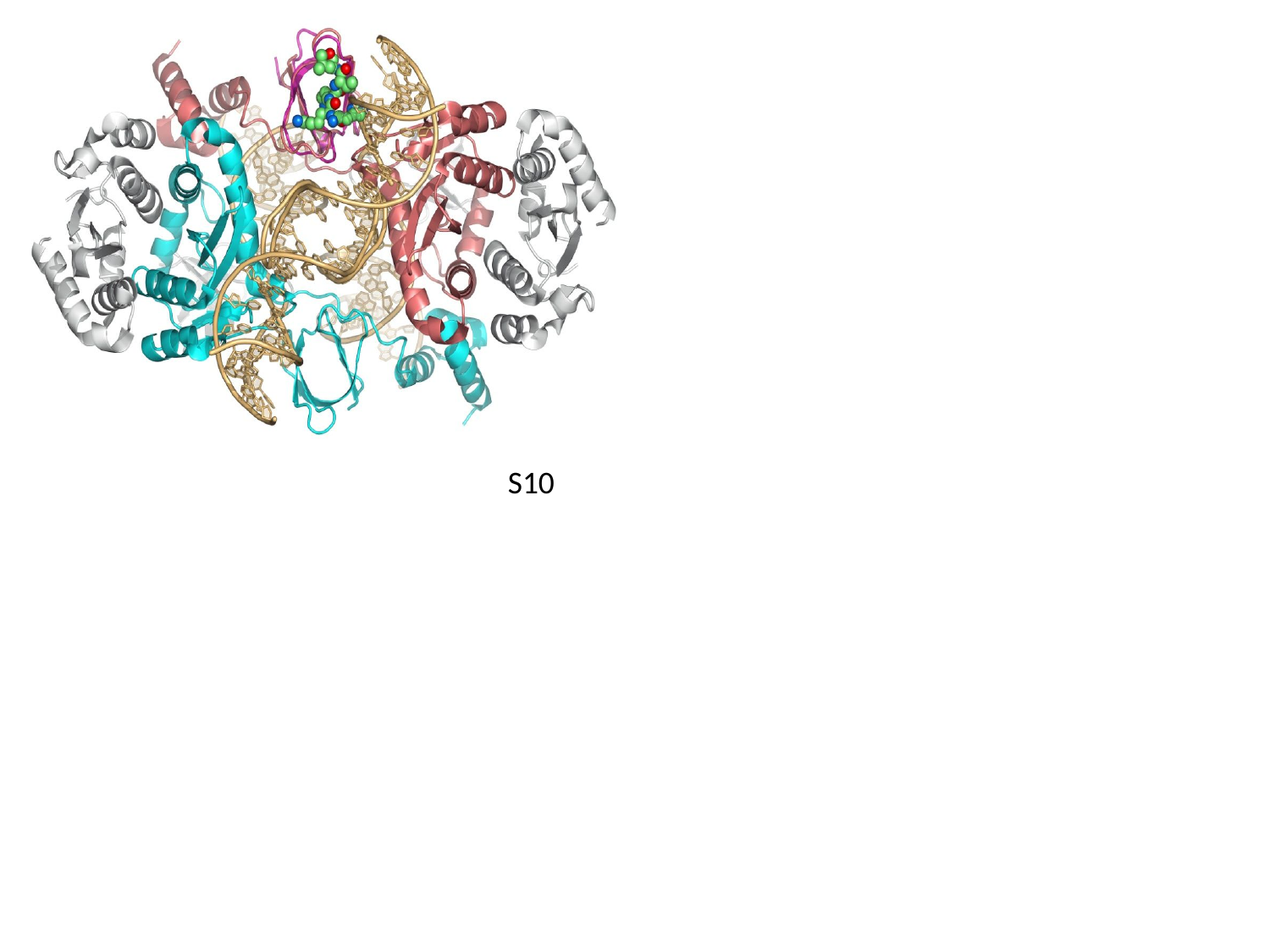

S10

## Slide 11
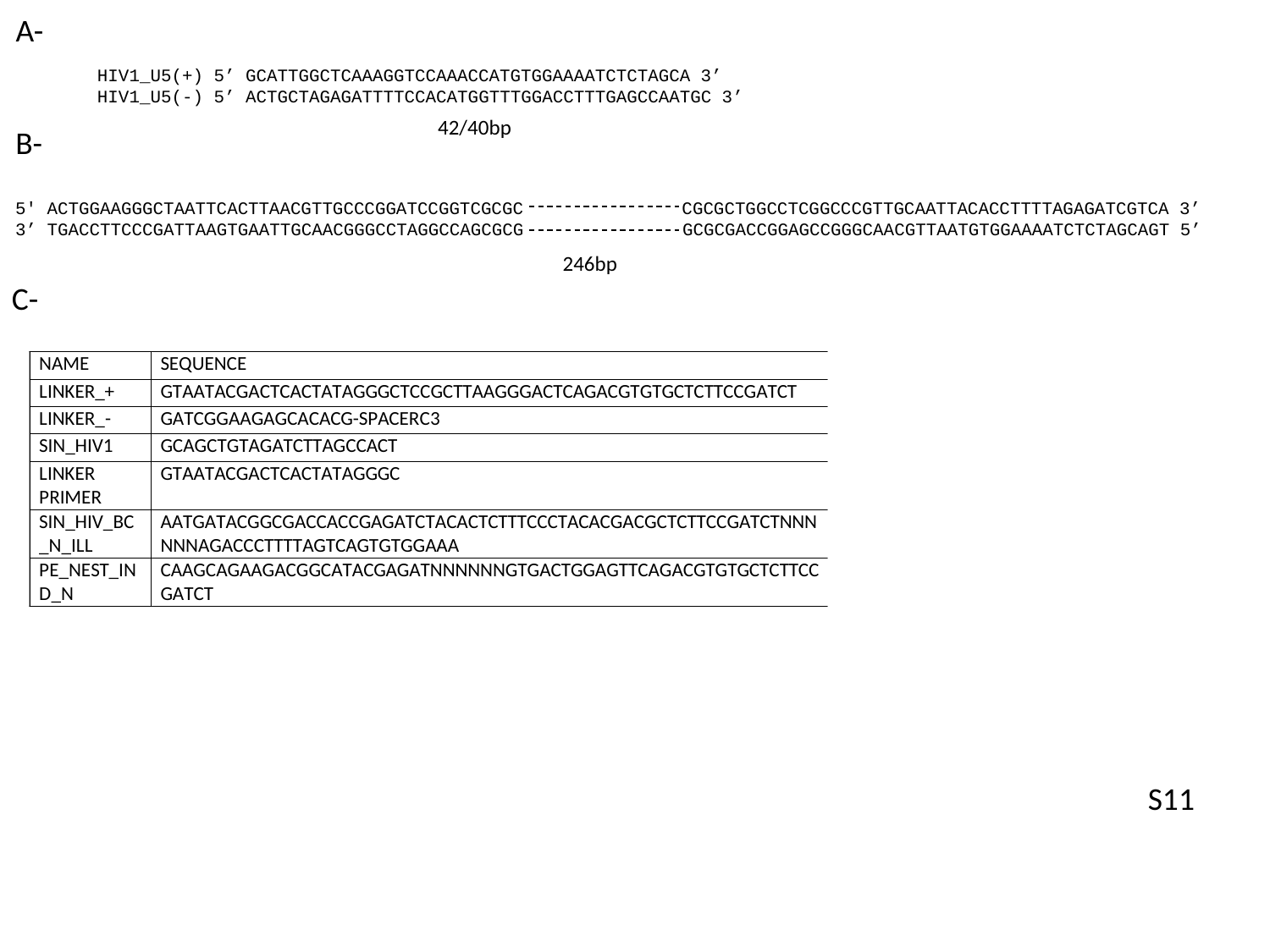

A-
HIV1_U5(+) 5’ GCATTGGCTCAAAGGTCCAAACCatgtggaaaatctctagca 3’
HIV1_U5(-) 5’ actgctagagattttccacatGGTTTGGACCTTTGAGCCAATGC 3’
42/40bp
B-
5' ACTGGAAGGGCTAATTCACTTAACGTTGCCCGGATCCGGTCGCGC CGCGCTGGCCTCGGCCCGTTGCAATTACACCTTTTAGAGATCGTCA 3’
3’ TGACCTTCCCGATTAAGTGAATTGCAACGGGCCTAGGCCAGCGCG GCGCGACCGGAGCCGGGCAACGTTAATGTGGAAAATCTCTAGCAGT 5’
246bp
C-
S11

## Slide 12
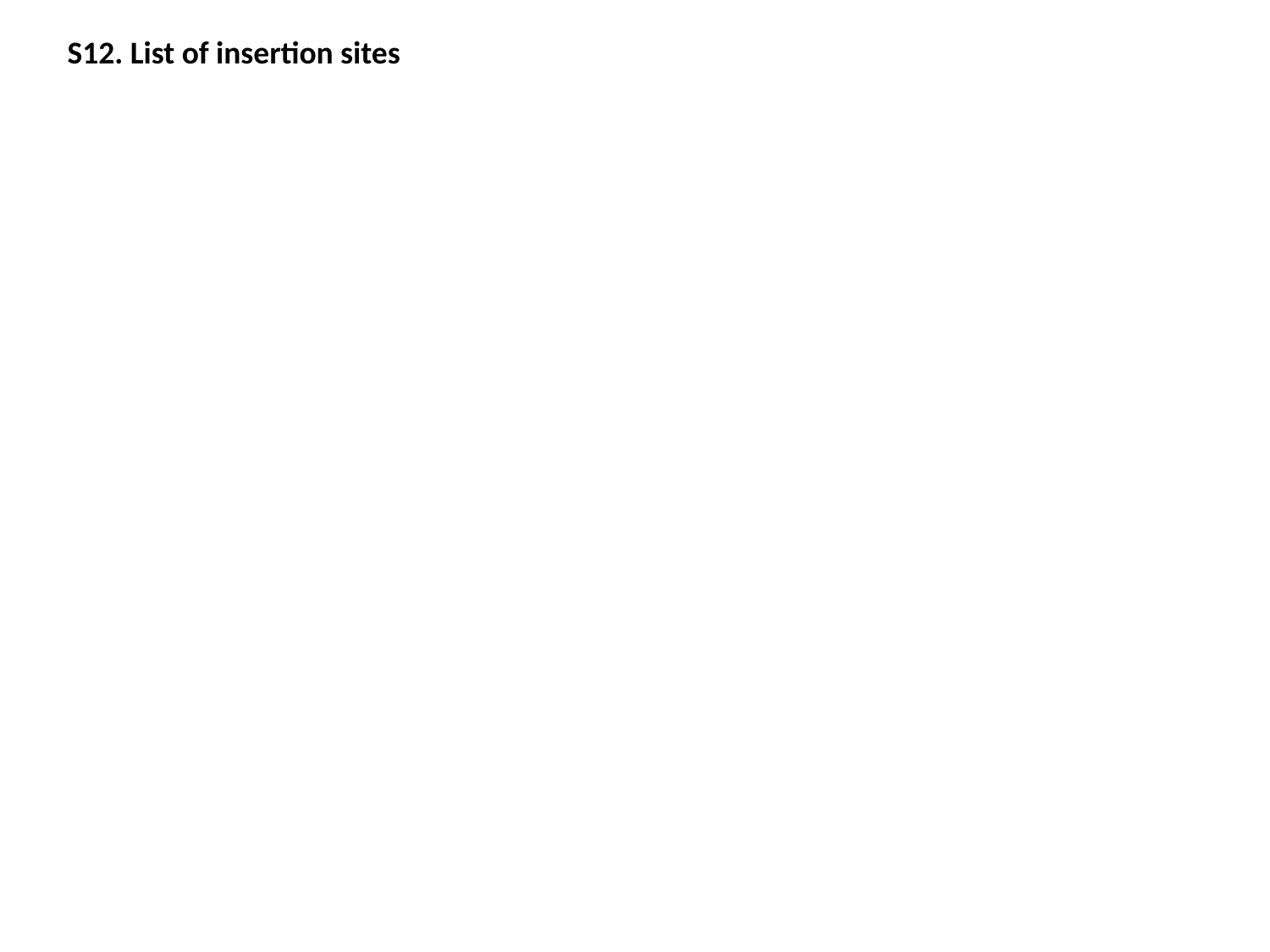

S12. List of insertion sites
